# Effect of *Mentha piperita* Essential Oil and Its Nanoemulsion on Microbial Growth, Physicochemical, and Organoleptic Properties of Mango Yogurt During Refrigerated Storage

**DOI:** 10.1002/fsn3.71845

**Published:** 2026-05-01

**Authors:** Fatemeh Chehri, Nafiseh Davati, Mostafa Karami

**Affiliations:** ^1^ Department of Food Science and Technology, Faculty of Food Industry Bu‐Ali Sina University Hamedan Iran

**Keywords:** essential oil, mango yogurt, *Mentha piperita*, nanoemulsion

## Abstract

Fruit yogurts are more susceptible to microbial contamination than plain yogurts due to the incorporation of fruit ingredients, increasing the need for natural preservatives. This study aimed to evaluate the effects of *Mentha piperita* essential oil (MEO) and *Mentha piperita* essential oil nanoemulsion (MPON) as effective natural preservatives on microbial growth, physicochemical properties, and sensory attributes of mango yogurt during refrigerated storage. Gas chromatography–mass spectrometry analysis identified menthol and l‐menthone as the major constituents of MEO. MPON exhibited significantly higher antioxidant activity (IC^50^ = 15 μg/mL) compared to MEO (IC^50^ = 40 μg/mL) and demonstrated enhanced antimicrobial efficacy against foodborne microorganisms. The results of minimum bactericidal concentration (MBC) and minimum inhibitory concentration (MIC) showed that *Pseudomonas aeruginosa* ATCC 9027 (MIC_MEO_ = 6000, MBC_MEO_ = 24,000, MIC_MPON_ = 2080, MBC_MPON_ = 4160 μg/mL) was the most resistant bacterium, and *Bacillus cereus* ATCC 11778 (MIC_MEO_ = 750, MBC_MEO_ = 1500, MIC_MPON_ = 520, MBC_MPON_ = 520 μg/mL) was the most sensitive. Incorporation of MEO, and particularly MPON, into mango yogurt significantly reduced microbial growth (*p* < 0.05), improved physicochemical stability during storage, and maintained desirable sensory properties. These findings indicate that a *Mentha piperita* essential oil nanoemulsion can serve as an effective natural preservative to enhance the overall quality and storage stability of fruit yogurts, supporting its potential application in dairy systems.

## Graphical abstract

Schematic illustration of “The effects of *Mentha piperita* essential oil and its nanoemulsion on microbial inhibition and quality preservation of mango yogurt during refrigerated storage”.

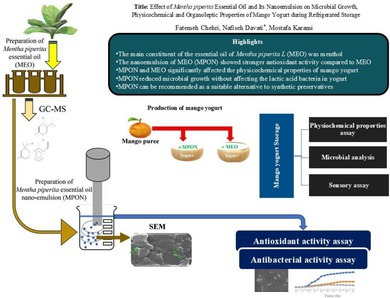

## 1 Introduction

Today, fruit yogurts have a large consumer market in the world due to their high biological properties (Jaster et al. 2018). The presence of fruits in yogurt can improve the taste, color, and texture of the product. In addition, their antioxidant compounds increase the nutritional value of yogurt (Massoud and Sharifan 2020; Ahmad et al. 2022). Mango is one of the most valuable fruits that can increase the nutritional value of fruit yogurt. Mango is a rich source of potassium, magnesium, vitamins C and A, and bioactive compounds such as mangiferin, carotenoids, flavonoids, phenolic acids, and fiber. Eating mangoes can relieve inflammation and promote the bifidobacterium growth in the gut (Ribeiro and Schieber 2010; Asuncion et al. 2023). The possibility of contamination with a variety of microorganisms is always higher in fruit yogurt than in plain yogurt due to the use of fruit ingredients (Jiang 2022). Therefore, the use of preservatives to control the microbial growth of this product is inevitable (Yildiz 2016). In recent decades, synthetic preservatives have been used in food processing to prevent the growth of microorganisms. However, the continuous use of these synthetic preservatives can increase the resistance of bacteria to them and cause some side effects for the consumer. Nowadays, due to consumers' desire for healthier and safer foods, the use of natural preservatives in foods is emphasized. Meanwhile, plant essential oils (EO) such as *Mentha piperita* EO (MEO) can be considered a suitable substitute for synthetic preservatives due to their antioxidant and antimicrobial properties (Kang et al. 2019; Falleh et al. 2021). Peppermint (*Mentha piperita* L.) belongs to the *Lamiaceae* family and is one of the most widely used EOs in the world due to its menthol and L‐menthone content. It has antifungal, antiviral, antibacterial, antioxidant, and anti‐inflammatory properties (Esmaeili et al. 2018; Barzegar et al. 2018; Yeşil et al. 2021). Numerous studies have shown that adding *Mentha piperita* can enhance the quality of dairy products like yogurt. For instance, Guemidi et al. (2024) studied the integration of phenolic extracts from *Mentha piperita* into yogurt and reported enhanced antioxidant properties, better physicochemical stability, and improved sensory properties during refrigeration. Azizkhani and Tooryan (2016) highlighted the antimicrobial benefits of peppermint, basil, and zataria in probiotic yogurts, which significantly decreased the presence of *Escherichia coli* and *Listeria monocytogenes* . However, there are always limitations in the use of EOs in the food industry due to low solubility in water, high volatility, sensitivity to heat, and unpleasant sensory properties (Liang et al. 2012; Yeşil et al. 2021). The solution to these problems is the encapsulation of EOs in the form of emulsion oil in water, such as nanoemulsions (particles with a diameter of 1–100 nm). The preparation of EOs in the structure of nanoemulsions can improve their dispersion in aqueous solutions, bioactivity, absorption, bioavailability, and stable release of the components, and consequently lead to an increase in their biological activities, such as antibacterial and antioxidant (Zhao et al. 2023). Despite the encouraging results, there is still a clear gap in research focusing specifically on nanoemulsified MEO. While encapsulation techniques have been successfully used with other EOs such as *Nepeta crispa* (mofarrah) in doogh (Haseli et al. 2023), *Zataria multiflora* Boiss L. EO nanoemulsion in Doogh (Shabani et al. 2025) and encapsulated oils to improve antioxidants in kefir (Tița et al. 2022), studies combining nanoemulsified peppermint oil with fruit yogurt are not yet accessible. Although MEO has a high potential for yogurt storage stability as a preservative, the use of a nanoemulsified form of MEO especially in fruit yogurt could lead to better quality and shelf life owing to its antimicrobial and flavor‐enhancing characteristics. The first objective of this study was to determine the chemical profile of MEO and assess the antioxidant and antimicrobial properties of both MEO and *Mentha piperita* essential oil nanoemulsion (MPON) against some food‐borne bacteria. The second objective was to determine the microorganism's growth, physicochemical, and organoleptic properties of mango yogurt with MEO and MPON compared to the control during refrigerated storage. Investigating this topic contributes to developing effective strategies for food preservation methods.

## 2 Materials and Methods

### 2.1 Materials

The MEO from the western region of Iran was purchased from Hakim Buali Sina Green Medicine Company (Hamadan, Iran) in Autom of 2023. Plain yogurt and mango were purchased from Pegah Company and a local market in Hamedan, respectively. All solvents, reagents, culture media, and chemical compounds were purchased from Sigma‐Aldrich Co., United States; Quelab Co., Canada; Merck Co., Darmstadt, Germany.

### 2.2 Gas Chromatography–Mass Spectrometry (GC–MS) Analysis

To better understand the composition of MEO, its chemical composition was characterized by GC–MS (Model 456‐SQ, Scion, Netherlands) equipped with a Cp Sil 5 capillary column (25 m × 0.25 mm, 0.25 μm film), following the analytical procedures of the Central Laboratory, Bu‐Ali Sina University, and according to Ardekani et al. (2019) and Moradi et al. (2023). Prior to analysis, the essential oil was diluted (1:10, v/v) in methanol, and 1.0 μL of the diluted sample was injected into the GC–MS system in split mode (1:10). Gas chromatography was set with an injector temperature of 290°C, helium as carrier gas flowing at 0.8 mL/min, and a column temperature program starting at 60°C (held for 1 min) and increasing to 280°C at 4°C/min (held for 10 min). For mass spectrometry, an electron current of 150 mA, an ionization energy of 70 eV, source and transfer line temperatures of 230°C, and a mass range of 50–500 m/z at a resolution of 0.7 were applied. The compounds were identified by GC–MS through comparison of their mass spectra with those in the NIST mass spectral library. The identified compounds are reported as compound name, retention time (RT), and relative abundance expressed as percentage of total peak area. The chromatogram and results of the analysis were submitted in File S1.

### 2.3 Preparation of MPON

To produce the nanoemulsion, the methods by Chu et al. (2020) and Moradi et al. (2023) were followed. To formulate the MPON, Tween 80 (7.5% v/v) as an emulsifier and MEO (7.5% v/v) as a dispersed phase were mixed in deionized water (85% v/v) as a continuous phase (800 rpm, 30 min). The mixture was then homogenized (OS20‐Pro, Dragon, China) at 12,000 rpm for 4 min and further processed using an ultrasonic homogenizer (SONOPULS HD 3100, BANDELIN, Germany; 25 kHz, 225 W) for 10 min. During ultrasonication, an ice bath was used to maintain the sample temperature and prevent thermal degradation of the essential oil components.

### 2.4 Particle Size Measurement

The polydispersity index (PDI) and average droplet size of MPON were measured at 25°C using a size analyzer, model Nano‐ZS ZEN3600 (Malvern, UK) (Liu et al. 2021). The span of MPON droplet sizes was determined using Equation (1).

(1) $$\text{Span}=\left(D90-D10\right)/D50$$

D10, D50, and D90 represent the size of MPON particles according to the curve of relative cumulative distribution of particle sizes at 10%, 50%, and 90% intensity.

### 2.5 Scanning Electron Microscope (SEM) of MPON

The size of MPON particles was characterized by SEM at 30.0 kV (FEI Model Quanta 450 FEG, USA).

### 2.6 Antioxidant Activity Assay

The antioxidant activity was measured by the potential of MEO and MPON in scavenging DPPH^1^ radicals (Singleton et al. 1999; Rashid et al. 2013). First, 50 μL each of different dilutions of MEO and MPON (0.01%, 0.1%, and 1%) were mixed with 5 mL of a 0.004% DPPH solution in methanol and then kept at 23°C for 30 min. The absorbance of the solutions compared to the control (including all reaction components without the test compound) was measured using a UV–VIS spectrophotometer (Model XD7500, LOVIBOND, Dortmund, Germany) at 517 nm. The percentage inhibition of DPPH by MEO and MPON was measured using Equation (2).

(2) $$\%\text{Inhibitory}=\frac{A0-A}{A0}\times 100$$

A0 = absorbance of the control A = absorbance of the test. IC^50^ refers to the concentration of an antioxidant required to scavenge 50% of DPPH free radicals.

### 2.7 Antibacterial Activity Assay

The antibacterial activity of MPON and MEO, i.e., minimum inhibitory concentration (MIC) and minimum bactericidal concentration (MBC), was assessed against *Pseudomonas aeruginosa* ATCC 9027, *E. coli* ATCC 25922, *Staphylococcus aureus* ATCC 29213, and *Bacillus cereus* ATCC 11778 using the broth microdilution method (Ferraro 2000; Moradi et al. 2023; Mashhadi et al. 2024). The food‐borne strains were cultured in Mueller‐Hinton broth (MHB) (Merck, Darmstadt, Germany) and incubated (37°C, 18 h). MEO and MPON were first dissolved in 99% DMSO and then serially diluted to obtain final concentrations of 0.37–48 mg/mL and 0.52–33.28 mg/mL in a 96‐well microplate for MEO and MPON, respectively. Each well of the microplate contained 100 μL of the MHB and 100 μL of each serially diluted MEO and MPON. Microbial inoculation of the microtiter plates was carried out with 1% (v/v) of overnight microbial cultures with a final count of approximately 10^6^ CFU/mL, followed by incubation (37°C, 24 h). The MIC is known as the lowest concentration of treatment that suppresses any visible growth of the microorganism in MHB. The positive control was the well containing 200 μL of bacterial suspension (1% v/v) in MHB without MEO or MPON. The negative control was the well containing 100 μL MHB and 100 μL of each serially diluted MEO and MPON. The bactericidal activity was tested by inoculating microtiter plates, which showed no visible growth, onto Mueller‐Hinton Agar (MHA) (Merck, Darmstadt, Germany). The MBC was the lowest treatment amount that completely stopped bacterial growth after 24 h at 37°C.

### 2.8 Time‐Kill Kinetics of MEO and MPON

The antibacterial activity of MEO and MPON on the growth curve of *P. aeruginosa* ATCC 9027, *E. coli* ATCC 25922, *B. cereus* ATCC 11778, and *S. aureus* ATCC 29213 was investigated (Foerster et al. 2016; Alayande et al. 2017). First, MEO and MPON in MBC were mixed in MHB, then inoculated with bacterial strains at 1% (v/v) with a final count of 10^6^ CFU/mL and subsequently incubated at 37°C. The control sample contained DMSO 1% (v/v) instead of MEO and MPON. The optical density of the inoculated MHB was measured for 12 h at intervals of 0.5 h using a UV–VIS spectrophotometer at 600 nm.

### 2.9 Antibacterial Mechanism of MEO and MPON

The morphological changes of the bacterial cells were assessed using an SEM (JSM‐840A, JEOL, Japan) according to Bajpai et al. (2013); Zhang et al. (2016). The MHB was inoculated with a culture of test bacteria (approximately 1 × 10^7^ CFU/mL) and then treated with MBC of MEO and MPON, followed by incubation at 37°C and 120 rpm for 3 h. After the incubation period, the cell pellets were separated using a centrifuge (1500 *g*, 10 min) and washed with 100 mM PBS^2^ (pH 7.4). The bacterial cells were fixed in 2.5% glutaraldehyde (4°C, 4 h). The cells were then dehydrated with 30%, 50%, 80%, 90%, and 100% ethanol, respectively. Finally, the dehydrated cells were sputter‐coated with the gold particles. Then the microscopic images were recorded with an SEM at a voltage of 12.0 kV.

### 2.10 Production of Mango Yogurt

First, the mangoes were washed, then peeled, and the cores removed. The mango was then pureed in a chopper for 30 s. The mango puree was then pasteurized (80°C, 10 min). Finally, the mango puree was produced with a Brix value of 25.4 and a pH value of 4.64. To produce the mango yogurt, 20% mango puree was first added to the plain yogurt. To produce yogurt in the Pegah factory, the milk was first heated to 65°C and homogenized. It was then pasteurized (90°C, 5 min) and cooled to 45°C. Subsequently, the starter culture containing *S. salivarius* *subsp. thermophilus* and *L. delbrueckii* *subsp. bulgaricus* (Chr. Hansen, Denmark) was added to the milk, which was then incubated at 45°C for 3 h until the acidity reached 60 Dornic degrees. The resulting yogurt was cooled at 0°C for 2 h and then transferred to cold storage at 4° *C. mango* yogurt was prepared in different formulations (as shown in Table 1). The mango yogurt samples were stirred with a stirrer (DragonLab OS20‐Pro, China) for 1 min and stored at 4°C for 25 days. Samples (in triplicate) were taken on day 0 (the first day of production), day 13, and day 25 to monitor changes over time.

**TABLE 1:** Different formulations of mango yogurt.

	Formulation
Y	Plain yogurt + 20% mango puree (control)
Y. E1	Plain yogurt + 20% mango puree + MEO^a^ in the final concentration of MIC^b^
Y. E2	Plain yogurt + 20% mango puree + MEO in the final concentration of MBC^c^
Y. N1	Plain yogurt + 20% mango puree + MPON^d^ in the final concentration of MIC
Y. N2	Plain yogurt + 20% mango puree + MPON in the final concentration of MBC

*Note:* MIC and MBC were selected for the most resistant bacteria, MIC_MEO_ = 6000, MBC_MEO_ = 24,000 and MIC_MPON_ = 2080, MBC_MPON_ = 4160 μg/mL.

^a^ *Mentha piperita* essential oil.

^b^ Minimum inhibitory concentration.

^c^ Minimum bactericidal concentration.

^d^ *Mentha piperita* essential oil nanoemulsion.

### 2.11 Physicochemical Properties of Mango Yogurt

Each sample was analyzed for fat content, pH, dry weight percentage (DW %), acidity, and protein content during the storage period according to the standard methods described by the Iran National Standards Organization (ISIRI) (ISIRI 2015, 2022a, 2022b, 2022c).

### 2.12 Syneresis

The syneresis of mango yogurt samples was analyzed according to the method described by Lastra‐Ripoll et al. (2023). 30 g of the sample was weighed and then centrifuged at 4000 rpm (15 min, 4°C). The supernatant was then separated and weighed. Syneresis was measured using Equation (3).

(3) $$\text{Syneresis}\;\left(\%\right)=\frac{\text{Supernatant weight}\;}{\;\text{Sample weight}}\times 100$$

### 2.13 Microbiological Analysis

Initially, 25 g of each yogurt sample was diluted with 225 mL of sterile Ringer's solution, followed by serial dilutions up to 10^−8^. After inoculation, plate count agar (PCA, Merck, Germany) plates were incubated at 37°C for 48 h to determine total bacterial counts and at 4°C for 10 days for psychrotrophic bacteria. De Man–Rogosa–Sharpe agar (MRS agar, Merck, Germany) was incubated at 42°C for 48 h for thermophilic lactic acid bacteria (LAB), and potato dextrose agar (PDA, Merck, Germany) was incubated at 25°C for 5 days for mold and yeast (ISIRI 2004; Amal et al. 2016; ISIRI 2022d; Kalori et al. 2023).

### 2.14 Sensory Properties Analysis

The sensory attributes of mango yogurts were evaluated after 25 days of cold storage. Fifty trained volunteers (25 women, 25 men, aged 20–40 years) participated in the study; panelists received prior training on evaluating yogurt sensory attributes. Samples were coded with random three‐digit numbers and served at room temperature (19°C–23°C). Sensory evaluations for overall acceptability, taste, texture, color, and odor were performed using a 5‐point hedonic scale (1 = lowest score, 5 = highest score). Evaluations were conducted in two sessions on the same day, with a 30–60 min interval between sessions to minimize sensory fatigue and carry‐over effects. Panelists were instructed to rinse their mouths with water between samples. Ethical approval was not required for the sensory tests of mango yogurts by our institute. All participants voluntarily participated in the sensory evaluation and provided informed consent before the study. The sensory evaluation was conducted in accordance with institutional ethical guidelines, and the authors declare no conflicts of interest.

### 2.15 Statistical Analysis

All data were statistically analyzed using a two‐way analysis of variance (ANOVA), with formulation and storage duration as treatments. Before interpreting the ANOVA results, model assumptions were checked by testing the normality of the residuals with the Shapiro–Wilk test and the homogeneity of variances with the Levene test. If both assumptions were met (*p* > 0.05), ANOVA was performed, followed by Bonferroni‐adjusted pairwise comparisons to detect significant differences between treatment groups. If any of the assumptions were violated (*p* ≤ 0.05), a non‐parametric aligned rank transformation (ART) was performed, followed by Bonferroni‐adjusted pairwise comparisons. For Mold, due to zero inflation and violation of model assumptions, the Kruskal‐Wallis test was used, followed by a Dunn post hoc test with Bonferroni adjustment. The results were expressed as means ± standard deviations of three repetitive measurements (Mean ± SD). For multiple comparisons, different letters were used in tables and figures to indicate statistically different groups. All statistical analyses were performed with the software R (version 4.5.0) using the packages ARTool, emmeans, multcompView, and PMCMRplus. Statistical significance was set at *p* < 0.05.

## 3 Results and Discussion

### 3.1 Chemical Profile of MEO

The results of the GC–MS analysis of MEO are shown in Table 2. In the present study, menthol (47.83%), L‐menthone (16.99%), benzofuran (7.75%), menthyl acetate (5.58%), limonene (4.9%), γ‐terpineol (4.14%), pulegone (3.11%), cyclohexanone (2.80%), and caryophyllene (1.48%) were the main constituents of MEO from western Iran. The results of the present study are consistent with those of Saharkhiz et al. (2012), Moghaddam et al. (2013), Beigi et al. (2018), Desam et al. (2019), Wardana et al. (2022). It is important to note that the composition of EOs such as MEO is affected by parameters such as the genetics of the plant, climate, soil type, growth phase at harvest, and pre‐ and post‐harvest handling (Yeşil et al. 2021).

**TABLE 2:** Chemical compositions of *Mentha piperita* essential oil.

Name	Retention time	% Of total
Menthol	11.48	47.83
L‐Menthone	10.57	16.99
Benzofuran	11.05	7.75
Menthyl acetate	15.23	5.58
Limonene	7.18	4.90
γ‐Terpineol	11.18	4.14
Pulegone	13.20	3.11
Cyclohexanone	10.84	2.80
Caryophyllene	19.50	1.48
β‐Pinene	5.83	0.64
Germacrene D	21.28	0.60
α‐Pinene	4.89	0.55
Piperitone	13.60	0.51
Butanoic acid	13.35	0.44
Levomenthol	11.76	0.39
Menthyl acetate	14.67	0.32
β‐Phellandrene	5.72	0.26
β‐Myrcene	6.13	0.25
L‐Menthol	11.19	0.21
Menthyl acetate	15.69	0.18
(‐)‐β‐Bourbonene	18.45	0.10
Caryophyllene oxide	24.02	0.15
1,6,10‐Dodecatriene	20.53	0.15
o‐Cymene	6.93	0.15
Tetrachloroethylene	2.71	0.13
Ledol	24.38	0.12
Terpineol	8.13	0.10
Total		99.83

### 3.2 The Droplet Size of MPON Particles

**FIGURE 1:**
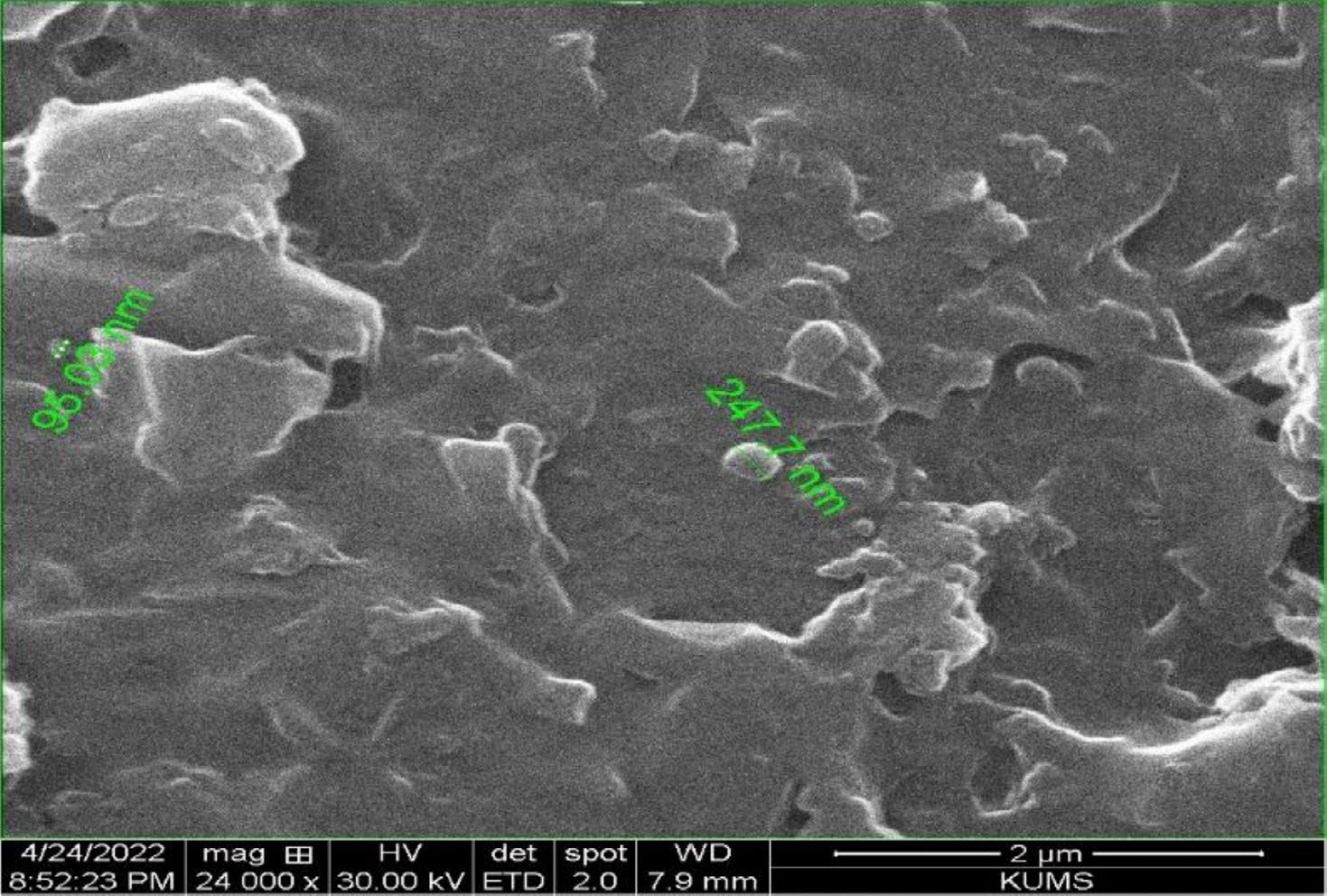
SEM image of *Mentha piperita* essential oil nanoemulsion prepared at 24,000× magnification.

In general, the ratio of surface area to volume increases as particle size decreases, especially at the nanoscale (Csicsák et al. 2023). Consequently, small particle sizes in nanoemulsion systems improve the solubility, dispersion, and permeability of hydrophobic compounds such as EOs (Algahtani et al. 2022). In delivery systems for bioactive compounds, nanoemulsion particles with an average droplet size between 10 and 200 nm, preferably smaller than 100 nm, have been proposed for more effective utilization of their antioxidant and antimicrobial activities (McClements and Li 2010; Donsì et al. 2012). Nanoemulsions in the particle size range of 10–200 nm exhibit remarkable kinetic stability and optical clarity, and improve the solubility and bioavailability of the encapsulated bioactive compounds (Singh et al. 2017; Roy et al. 2022). Consequently, this size provides effective antimicrobial and antioxidant activity, shelf‐life extension, and low sensory impact in food formulations. Droplets larger than 200 nm can be categorized as microemulsion or coarse emulsion, which reduces biological efficacy and increases the risk of phase separation. In addition, studies show that nanoemulsions below 100 nm resist phase separation when stored under cold conditions and promote long‐term physical stability (Pongsumpun et al. 2020). In the current study, Tween 80 was used as a surfactant to produce an oil‐in‐water emulsion because of the high equilibrium capacity of its hydrophilic–lipophilic balance. Decreasing the droplet size enhances the biological effectiveness of the lipophilic compounds encapsulated in the nanoemulsions by facilitating the movement of the active molecules through the cell membranes and increasing the reactivity of the droplets (Donsì and Ferrari 2016). In the current study, the polydispersity index (PDI) and the average droplet diameter of MPON were measured to be 0.296 and 95.10 nm, respectively. In nanoemulsion formulations, and in particular those containing EOs, homogeneity in terms of droplet size distribution is an important parameter quantified by the polydispersity index (PDI). A low PDI close to 0 indicates increased monodispersity, while values close to 1 reflect a broader size distribution, which may have a detrimental effect on the efficacy of the emulsion. Therefore, a high polydispersity value means a low consistency and indicates a lower uniformity in the size of the nanoemulsion droplets. The polydispersity index (PDI) was calculated as the ratio between the standard deviation and the average particle diameter according to Jaiswal et al. (2015) and Moradi and Barati (2019). The PDI of 0.296, as in the current study, is ideal for stable nanoemulsions. Such an emulsion indicates high physical stability, bioavailability, and functional efficacy of the EOs delivery system, and the risk of phase separation or coalescence is minimal. High stability of MPON is critical for its potential applications in antimicrobial delivery, food preservation, and drug formulation. In addition, the formation of MPON droplets on the nanometer scale (95.03 nm) was observed using SEM (Figure 1). In previous research, the average droplet size of the nanoemulsion of EOs was found to be 106.1 nm for *Cuminum cyminum* (Moradi et al. 2023), 11.8 nm for black pepper (Nie et al. 2023), 204.4 nm for sage (Yazgan 2020), less than 101 nm for peppermint and myrtle (Falleh et al. 2021), 64.60 nm for lemon (Liu et al. 2022), 107.50 nm for *Zataria multiflora* Boiss L. (Shabani et al. 2025), and 82.5–125.5 nm for thyme (Xue et al. 2015).

### 3.3 Antioxidant Activity of MEO and MPON

The antioxidant effect of MEO and its nanoemulsion (MPON) was tested using the DPPH radical scavenger and reported via the IC^50^ values. The IC^50^ values of MEO and MPON were 40 and 15 μg/mL, respectively. The antioxidant capacity of the EOs is mainly attributed to their high phenolic content. Plant materials contain a broad spectrum of phenolic compounds, including anthocyanins, phenolic acids, tannins, catechins, and flavonoids, which have an antioxidant effect (Kumar and Pandey 2013). In previous studies, the IC^50^ value (μg/mL) of the EO of Mentha species was reported to be 60.41 (Kızıl et al. 2010), 3.08 (Snoussi et al. 2015), and 2.53 (Mimica‐Dukić et al. 2003). Consistent with earlier findings, the antioxidant property of MEO in this study can be associated with its main constituents, including menthol (Ruberto and Baratta 2000; Mimica‐Dukić et al. 2003; Schmidt et al. 2009), L‐menthone (Mimica‐Dukić et al. 2003), benzofuran (Chand et al. 2017), and menthyl acetate (Mimica‐Dukić et al. 2003). As expected in this study, the EO nanoemulsion (MPON) showed a stronger antioxidant activity.

The superior performance of MPON may result from its smaller droplet size and enhanced solubility, which improves its ability to neutralize free radicals (Lou et al. 2017). In contrast, EOs do not mix well in aqueous solutions (Dhifi et al. 2016), thereby limiting their antioxidant potential compared with nanoemulsified systems (Sharifi and Sharifi 2023).

### 3.4 Antibacterial Activity of MEO and MPON

The antimicrobial activity of MEO and MPON against food‐borne bacteria was confirmed, as shown in Table 3. The results of antibacterial activity (MIC and MBC) showed that the tested Gram‐negative bacteria were more resistant compared to the tested Gram‐positive bacteria, so that *P. aeruginosa* (MIC_MEO_ = 6000, MBC_MEO_ = 24,000, and MIC_MPON_ = 2080, MBC_MPON_ = 4160 μg/mL) was the most resistant bacterium, and *B. cereus* (MIC_MEO_ = 750, MBC_MEO_ = 1500, and MIC_MPON_ = 520, MBC_MPON_ = 520 μg/mL) was the most sensitive bacterium. The phenolic compounds alter the permeability of the cell wall by interfering with the activity of enzymes related to energy production and disrupting the motive force of the protein, which in turn leads to cell death (Mishra, Singh, et al. 2020). The high antimicrobial effect of MEO can be attributed to its main chemical compounds, including menthol and L‐menthone (Liang et al. 2012; Desam et al. 2019); Wardana et al. (2022), menthyl acetate, and limonene (Liang et al. 2012). Previous studies indicate that EOs exhibit stronger antibacterial effects against Gram‐positive bacteria than Gram‐negative strains (Chao et al. 2000; Burt 2004; Mumivand et al. 2019; Moradi et al. 2023). The greater resistance of some gram‐negative bacteria to the antibacterial effect of EOs may be due to their cell wall having an outer membrane in addition to the lipoprotein‐peptidoglycan structure, which prevents the distribution of hydrophobic molecules across the hydrophilic layer (Garvey et al. 2011; Kourtesi et al. 2013; Cole et al. 2014; Elshafie et al. 2016). Gram‐negative bacteria also exhibit overexpression of efflux pumps underlying innate antimicrobial resistance, such as the AcrAB‐TolC efflux system (Garvey et al. 2011; Aelenei et al. 2016). In addition, the results of the current study showed that the nanoemulsion of *Mentha piperita* EO was more effective than pure EO against foodborne bacteria. The primary mechanism of EOs is the disruption and fusion of bacterial cell membranes (Valgas et al. 2007). Their nanoemulsions lead to higher membrane permeability, increased cytoplasmic leakage, and greater degradation of bacterial cell membrane integrity compared to pure EOs (Zhang et al. 2009). This finding is consistent with the results of Donsì et al. (2012); Desam et al. (2019); Falleh et al. (2021); Pajohi Alamoti et al. (2022); Mehraban et al. (2022); Moradi et al. (2023). However, it should be noted that the effectiveness of the antimicrobial activity of EOs may vary depending on the type of EO and the microbe tested. There are reports that some EOs have a stronger antimicrobial effect against gram‐negative bacteria than against gram‐positive bacteria (Prabuseenivasan et al. 2006; Patterson et al. 2019; Mishra, Devkota, et al. 2020). Therefore, EOs may show a different response to gram‐negative and gram‐positive bacteria.

**TABLE 3:** MIC^a^ and MBC^b^ of MEO^c^, and MPON^d^ against food‐borne bacteria.

Microorganisms	MEO (μg/mL)		MPON (μg/mL)	
	MIC	MBC	MIC	MBC
*S. aureus*	750	1500	520	1040
*B. cereus*	750	1500	520	520
*E. coli*	1500	6000	520	1040
*P. aeruginosa*	6000	24,000	2080	4160

^a^ Minimum inhibitory concentration.

^b^ Minimum bactericidal concentration.

^c^ *Mentha piperita* essential oil.

^d^ *Mentha piperita* essential oil nanoemulsion.

### 3.5 Kinetics of Antibacterial Activity of MEO and MPON

**FIGURE 2:**
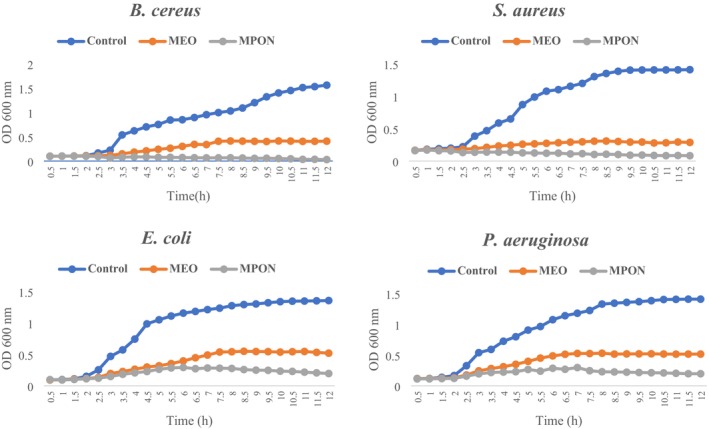
Growth curves of food‐borne bacteria affected by MEO and MPON at MBC in comparison with the control. MEO, *Mentha piperita* essential oil; MBC, minimum bactericidal concentration; MPON, *Mentha piperita* essential oil nanoemulsion.

MPON and MEO showed an inhibitory effect on the growth curves of foodborne bacteria when their MBC was applied compared to the control (Figure 2). Moreover, the inhibitory effect of MPON on microbial growth was more effective than that of MEO. According to Figure 2, the gram‐positive bacteria, *B. cereus* (with a logarithmic phase up to 10 h and a maximum OD of 0.413) and *S. aureus* (with a logarithmic phase up to 8.5 h and a maximum OD of 0.308) grew slowly at the MBC concentration of MEO and showed no significant growth at the MBC concentration of MPON. However, the gram‐negative bacteria, *E. coli* (with a logarithmic phase up to 8.5 h and a maximum OD of 0.549) and *P. aeruginosa* (with a logarithmic phase up to 8 h and a maximum OD of 0.529) grew slightly more than the two Gram‐positive bacteria at the MBC concentrations of MPON and MEO, so that *E. coli* and *P. aeruginosa* were more resistant than *B. cereus* and *S. aureus* . The observed reduction in viable cell counts in MPON‐treated media, compared with MEO, may be explained by the increased surface contact area and enhanced bioavailability of the active components, facilitating better penetration through bacterial cell walls. These results are consistent with previous studies by Liang et al. (2012), Moradi et al. (2023), Zhao et al. (2023), Shabani et al. (2025).

### 3.6 Morphology of Bacterial Cells Affected by MEO and MPON

**FIGURE 3:**
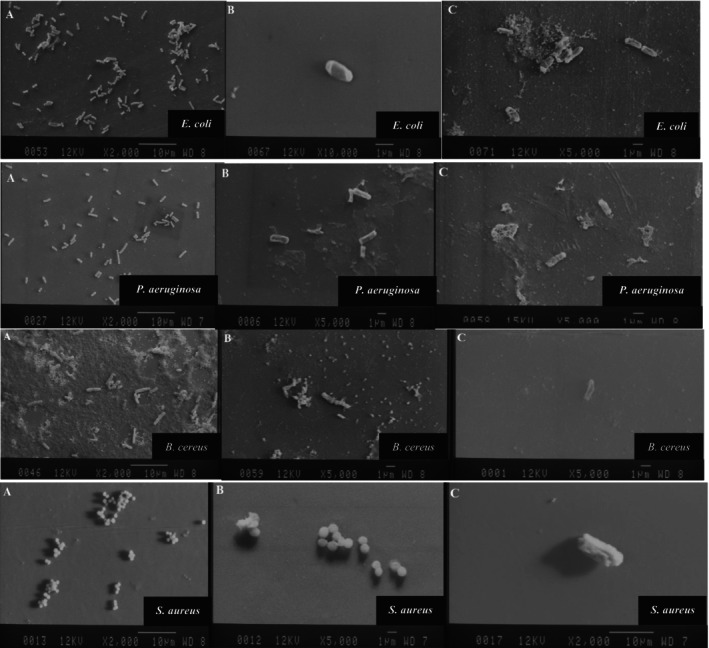
SEM images of food‐borne bacteria affected by MEO (B) and MPON (C) at MBC in comparison with the control (A) prepared at 2000–10,000× magnification. MBC, minimum bactericidal concentration; MEO, *Mentha piperita* essential oil; MPON, *Mentha piperita* essential oil nanoemulsion.

The phenolic compounds accumulate in the membrane due to their lipophilic properties, which leads to an increased disruption of membrane activity, damages the fatty acid, and disrupts the structure of the cell wall (Donsì et al. 2012; Kaur et al. 2020). The results of the SEM (Figure 3 and Figure S1) show that the cell walls of foodborne bacteria affected by MEO and MPON in MBC were severely damaged and had a wrinkled surface compared to the control cells, which had an intact and normal cell wall surface. More specifically, cells exposed to MBC of MEO lost their surface structure and showed signs of damage such as shrinkage, cracking, and lysis, while they disintegrated to a greater extent in MBC of MPON. The normal spherical structure of *S. aureus* cells also changed into an elongated and wrinkled rod structure under the MBC of MPON. The stronger destructive effect of the EO nanoemulsion compared to the pure EO was observed. Similarly, several studies have demonstrated the damaging effect of EOs and EO nanoemulsions on the cell wall, morphology, and cell death in *S. aureus* and *E. coli* affected by the peppermint oil nanoemulsion (Liu et al. 2021) and *Zataria multiflora* Boiss L. EO nanoemulsion (Shabani et al. 2025), in *E. coli* and *Salmonella typhi* affected by mustard EO (Turgis et al. 2009), *E. coli* affected by *Thymus daenensis* EO nanoemulsion (Moghimi et al. 2016), *Pseudomonas deceptionensis* affected by cinnamon EO nanoemulsion (Zhao et al. 2023), and *S. aureus* affected by peppermint EO (Kang et al. 2019).

### 3.7 Physicochemical Properties of Mango Yogurts

As shown in Table 4, significant differences (*p* < 0.05) were observed in the pH and titratable acidity of mango yogurt samples during refrigerated storage. Changes in acidity followed an inverse trend to pH variations throughout the storage period. The results indicated that the pH of all samples decreased from day 0 to day 13, which was accompanied by an increase in titratable acidity. From day 13 to day 25, a slight increase in pH and a corresponding decrease in acidity were observed in all samples. The initial decrease in pH was likely caused by LAB and yeasts degrading substrates and releasing organic acids during fermentation. Thereafter, the pH increased by the end of the storage period for each yogurt sample, probably due to the reduction of carbon sources, the consumption of protein compounds, and the antimicrobial effect of the MEO and MPON at high concentrations, which controls microbial growth (Azizkhani and Parsaeimehr 2018; Yangilar and Yildiz 2018; Kalori et al. 2023). In each storage period, the pH was lowest in the sample containing the MBC of MPON and highest in the control sample, and the pH decreased with increasing concentration of MEO and MPON from MIC to MBC. According to Razaei et al. (2013), the peppermint EO and its nanoemulsion probably slightly affected the pH of the samples due to their acidic properties. In addition, according to Chao et al. (2000), Azizkhani and Parsaeimehr (2018), LAB are more resistant to essential oils than other bacteria, and under certain conditions, their growth may be slightly stimulated, resulting in a further decrease in pH during the early storage period. Hence, in the current study, higher concentrations of MEO and MPON likely enhanced acid production by starter bacteria mainly during the initial phase of storage (up to day 13), which is consistent with the observed increase in acidity during this period. The pH changes pattern observed in yogurt samples in the current study is consistent with Razaei et al. (2013), Perina et al. (2015), Azizkhani and Parsaeimehr (2018), Yangilar and Yildiz (2018), Elama et al. (2019). As shown in Table 4, the percentage of dry weight increased over time, although the difference between the formulations was not significant. The main reasons for the increase in DW % of yogurt are the loss of water and gel restructuring during storage. Moreover, the other reasons for the increase in DW % of fruit yogurt can be fermentation, production, and storage conditions, and the effect of the hydrocolloids of the fruit on the texture of the yogurt (Bulgaru et al. 2021). During storage, the LAB ferments lactose and produces lactic acid, which leads to a decrease in pH and subsequently to greater coagulation of the proteins and an increase in DW % (Güler‐Akın and Akın 2007). This finding is in line with the results of Ismail et al. (2006), Güler‐Akın and Akın (2007), Bulgaru et al. (2021). In terms of fat content, neither the yogurt formulation nor the storage time had a significant effect (*p* > 0.05). The Y. E2 had the highest fat content (4.05%), likely due to the higher concentration of EO in this sample. No significant changes (*p* > 0.05) in protein levels were observed among treatments during refrigerated storage.

**TABLE 4:** Physicochemical properties of mango yogurt samples.

Sample formulation		pH	Acidity	Fat	DW %	Protein
Y	Day 0	4.35 ± 0.03^F^	1.00 ± 0.03^E^	3.96 ± 0.07^A^	16.26 ± 0.04^A^	3.26 ± 0.01^A^
	Day 13	4.29 ± 0.02^EF^	1.10 ± 0.02^AB^	3.96 ± 0.01^A^	16.80 ± 0.04^A^	3.25 ± 0.02^A^
	Day 25	4.30 ± 0.02^EF^	1.08 ± 0.03^ABC^	3.96 ± 0.01^A^	17.35 ± 0.04^A^	3.24 ± 0.02^A^
Y. E1	Day 0	4.30 ± 0.03^EF^	1.03 ± 0.01^E^	4.03 ± 0.01^A^	16.30 ± 0.04^A^	3.26 ± 0.00^A^
	Day 13	4.25 ± 0.03^DE^	1.12 ± 0.02^AB^	4.03 ± 0.01^A^	16.76 ± 0.04^A^	3.25 ± 0.02^A^
	Day 25	4.28 ± 0.02^EF^	1.09 ± 0.01^ABC^	4.00 ± 0.03^A^	17.33 ± 0.04^A^	3.24 ± 0.01^A^
Y. E2	Day 0	4.30 ± 0.03^EF^	1.04 ± 0.02^AB^	4.05 ± 0.01^A^	16.28 ± 0.04^A^	3.26 ± 0.02^A^
	Day 13	4.13 ± 0.02^B^	1.32 ± 0.02^CDE^	4.05 ± 0.00^A^	16.77 ± 0.04^A^	3.25 ± 0.02^A^
	Day 25	4.15 ± 0.02^bc^	1.26 ± 0.03^CDE^	4.05 ± 0.03^A^	17.31 ± 0.04^A^	3.24 ± 0.03^A^
Y. N1	Day 0	4.31 ± 0.01^EF^	1.03 ± 0.01^CDE^	3.98 ± 0.05^A^	16.29 ± 0.04^A^	3.26 ± 0.03^A^
	Day 13	4.20 ± 0.02^CD^	1.21 ± 0.03^BCD^	3.98 ± 0.00^A^	16.79 ± 0.04^A^	3.25 ± 0.03^A^
	Day 25	4.27 ± 0.02^DE^	1.16 ± 0.02^BCD^	3.98 ± 0.05^A^	17.34 ± 0.04^A^	3.23 ± 0.01^A^
Y. N2	Day 0	4.27 ± 0.03^DE^	1.14 ± 0.03^A^	3.99 ± 0.00^A^	16.29 ± 0.04^A^	3.26 ± 0.01^A^
	Day 13	3.98 ± 0.04^A^	1.54 ± 0.01^E^	3.99 ± 0.00^A^	16.79 ± 0.04^A^	3.24 ± 0.02^A^
	Day 25	4.00 ± 0.05^A^	1.47 ± 0.02^DE^	3.99 ± 0.00^A^	17.34 ± 0.04^A^	3.23 ± 0.01^A^

*Note:* Different letters indicate statistically significant differences (*p* < 0.05) in each column (*n* = 3).

Abbreviations: MBC, minimum bactericidal concentration; MEO, *Mentha piperita* essential oil; MIC, minimum inhibitory concentration; MPON, *Mentha piperita* essential oil nanoemulsion; Y. E1, Plain yogurt + 20% mango puree + MEO in the final concentration of MIC; Y. E2, plain yogurt + 20% mango puree + MEO in the final concentration of MBC; Y. N1, plain yogurt + 20% mango puree + MPON in the final concentration of MIC; Y. N2, plain yogurt + 20% mango puree + MPON in the final concentration of MBC; Y, plain yogurt + 20% mango puree (control).

### 3.8 Syneresis of Mango Yogurts

Syneresis, i.e., the separation of whey from yogurt, occurs when the three‐dimensional structure of the yogurt gel is deformed and weakened, resulting in the yogurt gel no longer retaining the serum (Saberi et al. 2023). In the current study, no syneresis was observed in the samples during storage, which could be due to the presence of pectin in the mango, which prevents syneresis in the mango yogurt. Pectin compounds can reversibly attach to casein and increase spatial repulsion, reducing the attachment of casein proteins (Amal et al. 2016). Pectin increases the viscosity of yogurt by thickening it and helps to stabilize the texture of yogurt by preventing syneresis (Lucey and Singh 1997). Our results are consistent with the findings of Arioui et al. (2017), Bulgaru et al. (2021), Mirazimi et al. (2022), Rahman et al. (2024).

### 3.9 Microbiological Analysis

**FIGURE 4:**
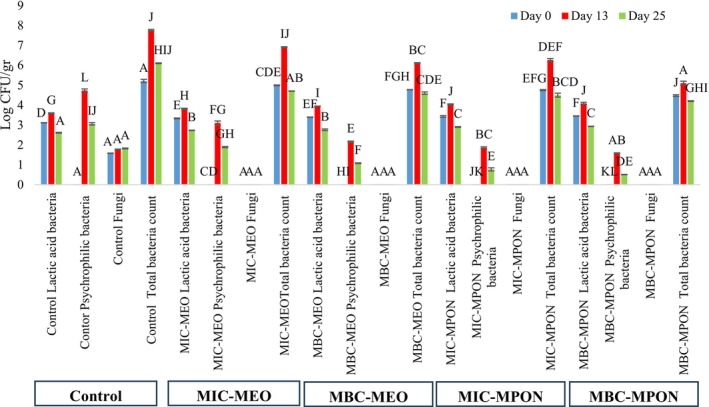
Effect of MPON and MEO on the microbial growth in mango yogurt samples during 25 days of storage period. Error bars represent the standard deviation of three repetitive measurements. Different letters indicate statistically significant differences (*p* < 0.05); MBC, minimum bactericidal concentration; MBC‐MEO, mango yogurt + Mentha *piperita* essential oil in the final concentration of MBC; MBC‐MPON, mango yogurt + *Mentha piperita* essential oil nanoemulsion in the final concentration of MBC; MEO, *Mentha piperita* essential oil; MIC, minimum inhibitory concentration; MIC‐MEO, mango yogurt + Mentha *piperita* essential oil in the final concentration of MIC; MIC‐MPON, mango yogurt + *Mentha piperita* essential oil nanoemulsion in the final concentration of MIC; MPON, *Mentha piperita* essential oil nanoemulsion.

In this study, we observed that the effects of MEO and MPON on the growth of microbial flora in yogurt varied according to their concentration and the type of microorganism, rather than producing a broad inhibitory effect. These compounds showed selective antimicrobial activity against the non‐lactic flora studied, while promoting the growth of lactic flora. As can be seen in Figure 4, the number of psychrotrophic bacteria, LAB, and total bacterial count changed significantly between the different samples and during the storage period (*p* < 0.05). The number of psychrotrophic bacteria, LAB, and total bacterial count increased until the 13th day of storage and then decreased until the 25th day of storage. The decrease in microbial growth at the end of the storage period could be due to the antagonistic effect of microorganisms, the reduction of nutrients, and unfavorable changes in environmental conditions for microbial growth. The results showed that at the sub‐lethal concentrations of MEO and MPON, the number of LAB increased in the mango yogurt samples compared to the control sample during each storage period. This effect may be attributed to the presence of phenolic compounds, flavonoids, and other bioactive molecules in peppermint essential oil, which may modulate the microbial environment and selectively support the viability of LAB. In addition, LAB possess adaptive mechanisms, including modifications in membrane composition, proton extrusion systems, and stress‐response pathways, enabling them to better tolerate antimicrobial compounds, whereas non‐lactic flora is generally more susceptible to membrane damage (Wang et al. 2018; Nguyen and Nguyen 2024). Azizkhani and Parsaeimehr (2018), Razaei et al. (2013), Yangilar and Yildiz (2018), Khalil and Hussain (2024), de Souza (2021) reported the stimulatory effect of *Mentha piperita* EO; *Mentha piperita* EO; ginger and chamomile; aromatic mint extract; peppermint EO on the growth of LAB, respectively. Since the psychrotrophic bacteria can grow at temperatures below 10°C, their numbers increased by the 13th day in the refrigerator. In addition, the number of psychrotrophic bacteria and total bacteria decreased significantly (*p* < 0.05) compared to the control sample when the concentration of MEO and MPON was increased, which could be due to their antimicrobial properties. Most EOs and plant extracts exert their antimicrobial activities by interfering with processes associated with the cell membrane of bacteria, including electron transfer, ion gradient, protein transport, and enzyme‐dependent reactions (Andrade‐Ochoa et al. 2021). During the 25‐day storage period, fungal growth was inhibited in the MEO and MPON samples compared to the control sample. The compounds of the EOs increase the permeability of the fungal membranes. The high menthol content in the EO of *Mentha piperita* disrupts the cell membranes of the fungi and leads to their death. In addition, EOs can control microbial growth more effectively when the pH of a food, such as yogurt, is low, as they dissolve better within the lipid layer of microbial membranes due to their hydrophobic nature in acidic environments. This probably helped to disrupt the cell membranes of the microbes, causing the contents to leak out and the cells to die, especially at low pH (Holley and Patel 2005; da Silva and Franco 2012). As mentioned earlier, it was expected that MPON could control the microbial growth of mango yogurt samples during cold storage more effectively than pure EO. Consequently, the presence of *Mentha piperita* L., especially at the nanoscale, effectively reduces the number of psychrotrophic bacteria, total bacterial count, and fungi compared to the control sample. The results of the microbial analysis of the current study are in line with Khusniati and Widyastuti (2008), Razaei et al. (2013), Moghaddam et al. (2013), Azizkhani and Tooryan (2015), Azizkhani and Parsaeimehr (2018), Desam et al. (2019), Fadavi et al. (2020), Milanović et al. (2021), Pajohi Alamoti et al. (2022), Mehraban et al. (2022).

### 3.10 The Sensory Characteristics of the Samples

**FIGURE 5:**
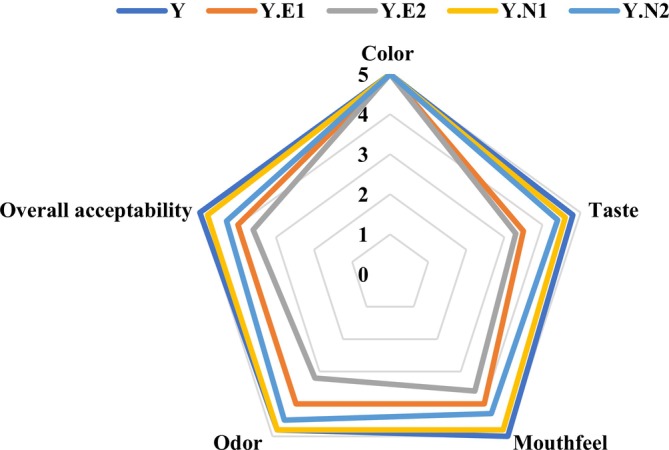
Consumer acceptability scores on a 5‐point scale for mango yogurt samples on the 25th day of storage. MBC, minimum bactericidal concentration; MEO, *Mentha piperita* essential oil; MIC, minimum inhibitory concentration; MPON, *Mentha piperita* essential oil nanoemulsion; Y, plain yogurt +20% mango puree (control); Y. E1, plain yogurt +20% mango puree + MEO in the final concentration of MIC; Y. E2, plain yogurt +20% mango puree + MEO in the final concentration of MBC; Y. N1, plain yogurt +20% mango puree + MPON in the final concentration of MIC; Y. N2, plain yogurt +20% mango puree + MPON in the final concentration of MBC.

The results of the sensory characteristics of the mango yogurt samples are shown in Figure 5. As can be seen in this Figure, the color of the mango yogurt samples was not affected by the concentration of EOs, so this value was the same for all samples. The highest score for mouthfeel (5–4.8), odor (4.8–4.8), taste (4.8–4.6), and overall acceptability (5–4.8) was obtained for the control and Y.N1 sample (mango yogurt with MIC concentration of MPON), respectively. The lowest score for mouthfeel (3.6), odor (3.2), taste (3.3), and overall acceptability (3.6) was obtained for Y.E2 (mango yogurt with MBC concentration of MEO) after 25 days of storage. The lower scores of sensory evaluations of sample Y.E2 could be due to the high concentration of *Mentha piperita* EO (24 mg/mL) compared to the other samples, which leads to undesirable sensory characteristics. However, the low concentration of *Mentha piperita* EO (Y.N1, 4.16 mg/mL) imparted a mild odor and taste to the mango yogurt, which increased the overall acceptability of the samples. Consequently, these results showed that the low concentration of *Mentha piperita* EO at the nanoscale effectively improved the properties of mango yogurt compared to other samples containing EO. It should be noted that the nanoemulsification of *Mentha piperita* EO likely played a key role in modulating the sensory characteristics of mango yogurt. The significant reduction in droplet size and improved dispersion of the oil phase within the matrix of the yogurt can enhance physical stability and create a more homogeneous microstructure. Such structural uniformity may contribute to a smoother mouthfeel and improved textural perception by reducing phase separation and minimizing oil droplet aggregation. Moreover, the finer droplet distribution can promote more uniform light scattering, potentially leading to improved color homogeneity. Nanoemulsions may allow more controlled release of volatile compounds by entrapping essential oil components within small droplets, thus reducing the intensity of sharp tastes and improving overall odor acceptability. These mechanistic aspects suggest that the observed improvements in sensory scores are closely linked to the physicochemical changes induced by nanoemulsification, as supported by previous studies on nanoemulsified flavor systems in food products. These results are in line with previous studies by Moghaddam et al. (2013), Moradi et al. (2023), Shabani et al. (2025).

## 4 Conclusion

In this study, the potential of *Mentha piperita* essential oil (MEO) and its nanoemulsion (MPON) as a natural preservative to enhance the quality and extend the shelf life of mango yogurt was demonstrated. The antioxidant and antimicrobial properties of MPON and MEO have been validated, likely attributed to the presence of menthol, L‐menthone, benzofuran, menthyl acetate, limonene, γ‐terpineol, and pulegone as the main constituents of MEO identified by GC–MS, whereas MPON showed superior efficacy due to its nanoscale formulation. SEM also confirmed the antimicrobial effect by demonstrating structural cell damage to the microorganisms. MPON had a drastically low IC^50^ value and was more effective in inhibiting fungi and regulating psychrotrophic and total microbial counts without affecting the viability of LAB. No specific restrictions that would substantially affect the outcomes were identified. However, it should be noted that the study was conducted under controlled experimental conditions. While this approach enhances internal consistency, it may not fully reflect the complexity of real or industrial environments, where additional sources of variability may influence the results. Consequently, the generalizability of the findings to broader conditions may be limited. Future research should focus on formulation optimization, long‐term stability evaluation, and assessment of the feasibility of large‐scale production and economic applicability. Such investigations would further support the practical implementation of this approach in dairy and functional food systems. In conclusion, the addition of MEO, preferably in the form of a nanoemulsion, can be recommended as a natural preservative to ensure microbiological stability, prevent the loss of sensory properties, and prolong the shelf life of foods such as fruit yogurts, paving the way for its use as a natural preservative.

## Author Contributions

**Fatemeh Chehri:** conceptualization, investigation, methodology, validation, visualization, software, formal analysis, data curation, resources. **Nafiseh Davati:** conceptualization, methodology, writing – review and editing, project administration, supervision, resources, data curation, writing – original draft, funding acquisition, investigation. **Mostafa Karami:** conceptualization, data curation.

## Funding

This research was supported by Bu‐Ali Sina University, Hamedan, Iran (Grant No. 40249).

## Conflicts of Interest

The authors declare no conflicts of interest.

## Supporting information


**Figure S1:** High‐resolution images corresponding to SEM results of food‐borne bacteria treated with MEO (B) and MPON (C) at the MBC, compared with the control (A).

**File S1:** Supporting Information.

## Data Availability

The data that supports the findings of this study are available in the Supporting Information of this article. Additional data will be made available on request.

## Endnotes

^1^
2,2′‐Diphenyl‐1‐picrylhydrazyl.

^2^
Phosphate buffer solution.
