# Supplementary material for: Effect of Mentha piperita Essential Oil and Its Nanoemulsion on Microbial Growth, Physicochemical, and Organoleptic Properties of Mango Yogurt During Refrigerated Storage
Source: Food Sci Nutr. 2026 May 1;14(5):e71845. doi: 10.1002/fsn3.71845 (PMC13135118; doi:10.1002/fsn3.71845)
Supplement: Supplementary file 1 — Figure S1: High‐resolution images corresponding to SEM results of food‐borne bacteria treated with MEO (B) and MPON (C) at the MBC, compared with the control (A). [file FSN3-14-e71845-s001.docx]

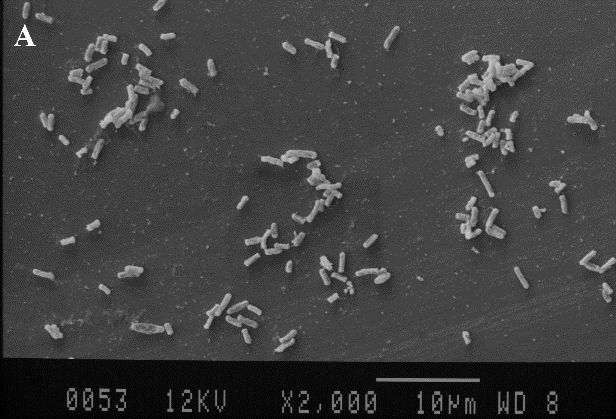

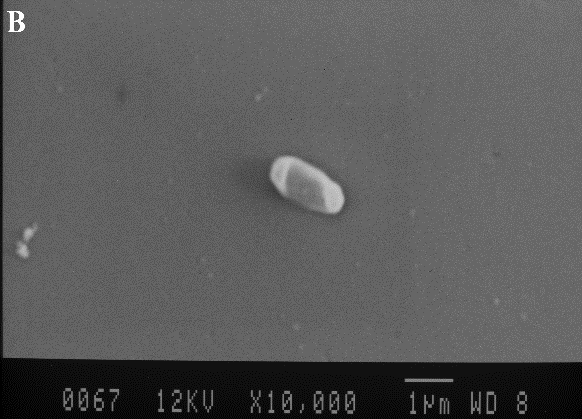

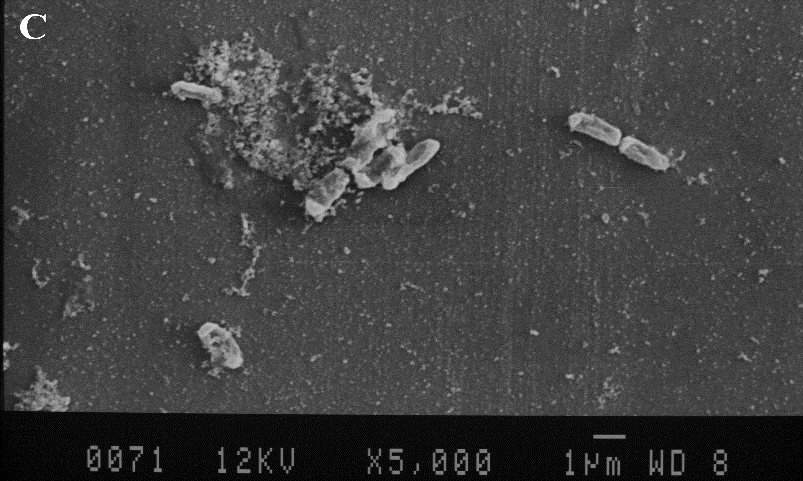


***E. coli***

***E. coli***

***E. coli***

**Supplementary Figure S1.** High-resolution images corresponding to SEM results of food-borne bacteria treated with MEO (B) and MPON (C) at the MBC, compared with the control (A).


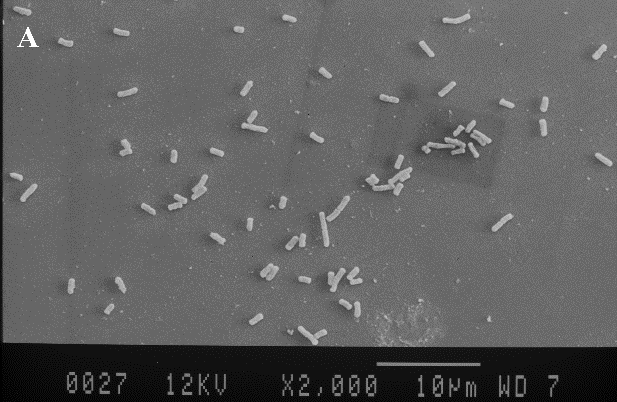

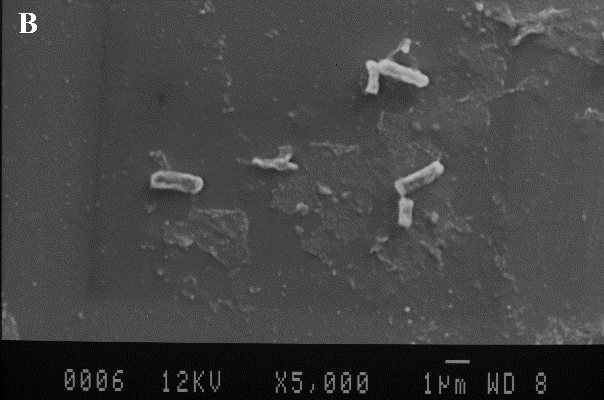

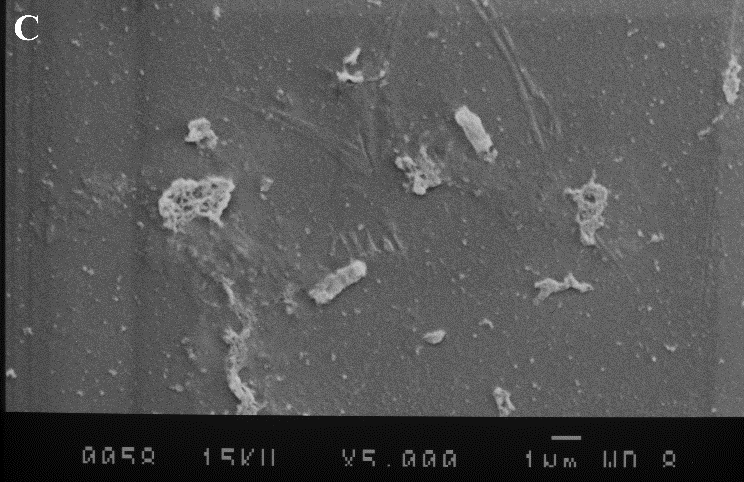


***P. aeruginosa***

***P. aeruginosa***

***P. aeruginosa***


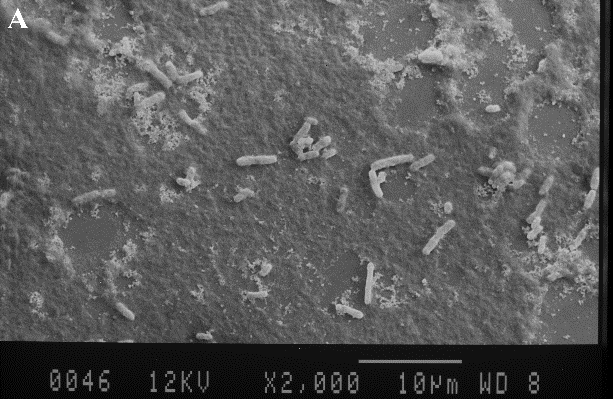

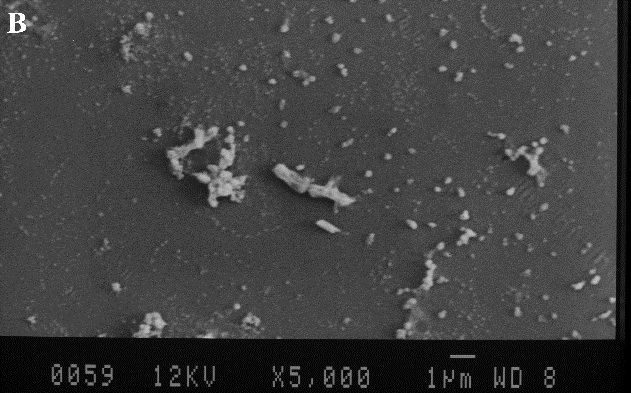

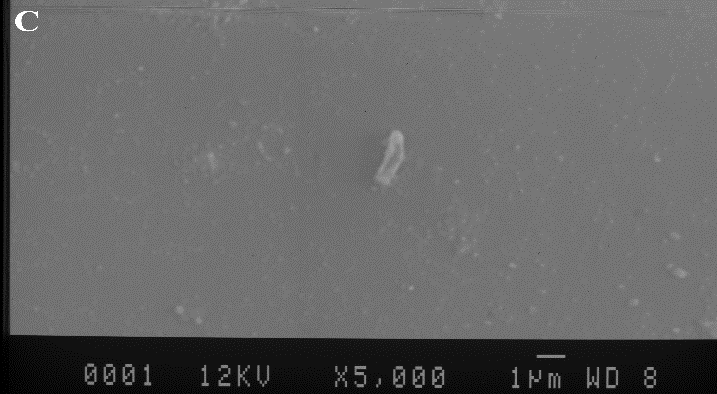


*B. cereus*

*B. cereus*

*B. cereus*


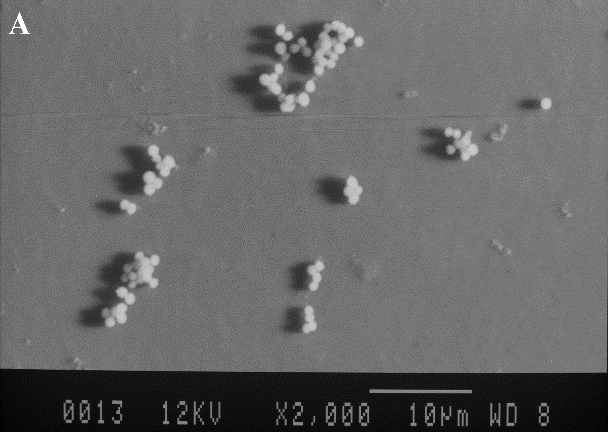

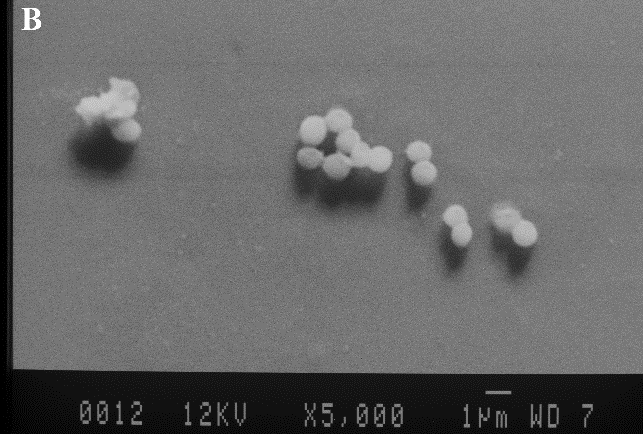

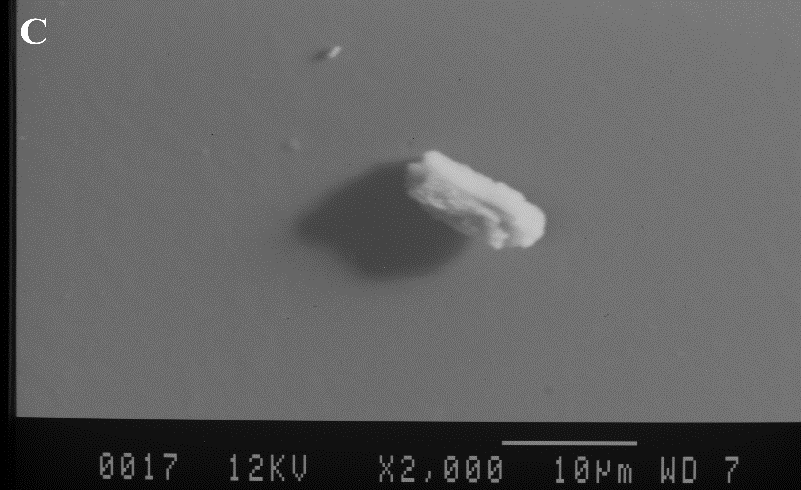


***S. aureus***

***S. aureus***

***S. aureus***
