# Supplementary material for: Effect of Mentha piperita Essential Oil and Its Nanoemulsion on Microbial Growth, Physicochemical, and Organoleptic Properties of Mango Yogurt During Refrigerated Storage
Source: Food Sci Nutr. 2026 May 1;14(5):e71845. doi: 10.1002/fsn3.71845 (PMC13135118; doi:10.1002/fsn3.71845)
Supplement: Supplementary file 2 — File S1: Supporting Information. [file FSN3-14-e71845-s002.zip › supplementary file 1/10.577.docx]

Hit 1 : l-Menthone

C10H18O; MF: 951; RMF: 968; Prob 28.2%; CAS: 14073-97-3; Lib: mainlib; ID: 80525.

112

O

69

41

55

139

39

97

43

83

154

95

27

53

51

67

81

93

125

100

50

0

20 30 40 50 60 70 80 90 100 110 120 130 140 150 160

(mainlib) l-Menthone

O

Name: l-Menthone Formula: C10H18O

MW: 154 Exact Mass: 154.135765 CAS#: 14073-97-3 NIST#: 114554 ID#: 80525 DB: mainlib

Other DBs: TSCA, HODOC, EINECS

Contributor: NIST Mass Spectrometry Data Center, 1990. Related CAS#: 21060-23-1

10 largest peaks:

112 999 | 69 794 | 41 660 | 55 589 | 139 427 | 70 356 | 97 332 | 39 302 | 154 294 | 111 291 |

Synonyms:

1.Cyclohexanone, 5-methyl-2-(1-methylethyl)-, (2S-trans)- 2.Cyclohexanone, 5-methyl-2-(1-methylethyl)-, (2S,5R)- 3.trans-(-)-p-Menthan-3-one

4.(2S,5R)-2-Isopropyl-5-methylcyclohexanone

Page 1 of 1
