# Supplementary material for: Effect of Mentha piperita Essential Oil and Its Nanoemulsion on Microbial Growth, Physicochemical, and Organoleptic Properties of Mango Yogurt During Refrigerated Storage
Source: Food Sci Nutr. 2026 May 1;14(5):e71845. doi: 10.1002/fsn3.71845 (PMC13135118; doi:10.1002/fsn3.71845)
Supplement: Supplementary file 2 — File S1: Supporting Information. [file FSN3-14-e71845-s002.zip › supplementary file 1/11.055.docx]

Hit 1 : Benzofuran, 4,5,6,7-tetrahydro-3,6-dimethyl-

C10H14O; MF: 923; RMF: 945; Prob 89.8%; CAS: 494-90-6; Lib: mainlib; ID: 76920.

108

O

150

79

27

39

43

51 55

65

91 95

105

115 121

135

100

50

0

20 30 40 50 60 70 80 90 100 110 120 130 140 150 160

(mainlib) Benzofuran, 4,5,6,7-tetrahydro-3,6-dimethyl-

O

Name: Benzofuran, 4,5,6,7-tetrahydro-3,6-dimethyl-Formula: C10H14O

MW: 150 Exact Mass: 150.1044655 CAS#: 494-90-6 NIST#: 21454 ID#: 76920 DB: mainlib

Other DBs: Fine, TSCA, HODOC, NIH, EINECS

10 largest peaks:

108 999 | 150 258 | 79 130 | 109 84 | 39 75 | 77 72 | 41 60 | 91 49 | 51 34 | 27 33 |

Synonyms:

1.p-Mentha-3,8-diene, 3,9-epoxy-2.Menthofuran

3.Menthofurane

4.3,9-Epoxy-p-mentha-3,8-diene

5.3,6-Dimethyl-4,5,6,7-tetrahydro-1-benzofuran #

Page 1 of 1
