# Supplementary material for: Effect of Mentha piperita Essential Oil and Its Nanoemulsion on Microbial Growth, Physicochemical, and Organoleptic Properties of Mango Yogurt During Refrigerated Storage
Source: Food Sci Nutr. 2026 May 1;14(5):e71845. doi: 10.1002/fsn3.71845 (PMC13135118; doi:10.1002/fsn3.71845)
Supplement: Supplementary file 2 — File S1: Supporting Information. [file FSN3-14-e71845-s002.zip › supplementary file 1/11.181.docx]

Hit 1 : Cyclohexanol, 1-methyl-4-(1-methylethyl)-

C10H20O; MF: 928; RMF: 941; Prob 15.0%; CAS: 21129-27-1; Lib: mainlib; ID: 35543.

71

OH

81

95

41

55

43

57 67

123

29

39

53

109

138

15

31

65

100

50

0

10 20 30 40 50 60 70 80 90 100 110 120 130 140 150 160 170

(mainlib) Cyclohexanol, 1-methyl-4-(1-methylethyl)-

OH

Name: Cyclohexanol, 1-methyl-4-(1-methylethyl)- Formula: C10H20O

MW: 156 Exact Mass: 156.151415 CAS#: 21129-27-1 NIST#: 75281 ID#: 35543 DB: mainlib

Other DBs: TSCA, HODOC, EINECS

Contributor: RADIAN CORP 10 largest peaks:

71 999 | 81 724 | 95 614 | 41 469 | 55 453 | 43 352 | 67 325 | 82 323 | 57 320 | 69 319 |

Synonyms:

1.1-Methyl-4-(1-methylethyl)cyclohexanol 2.4-Isopropyl-1-methylcyclohexanol

3.γ-Terpineol, dihydro- 4.p-Menthan-1-ol

Page 1 of 1
