# Supplementary material for: Effect of Mentha piperita Essential Oil and Its Nanoemulsion on Microbial Growth, Physicochemical, and Organoleptic Properties of Mango Yogurt During Refrigerated Storage
Source: Food Sci Nutr. 2026 May 1;14(5):e71845. doi: 10.1002/fsn3.71845 (PMC13135118; doi:10.1002/fsn3.71845)
Supplement: Supplementary file 2 — File S1: Supporting Information. [file FSN3-14-e71845-s002.zip › supplementary file 1/11.487.docx]

Hit 2 : Cyclohexanol, 5-methyl-2-(1-methylethyl)-, (1α,2β,5α)-(±)-

C10H20O; MF: 963; RMF: 963; Prob 17.1%; CAS: 15356-70-4; Lib: replib; ID: 8667.

71

81

OH

95

41

55

43

57

67

123

29

39

138

53

109

15

31

65

155

100

50

0

10 20 30 40 50 60 70 80 90 100 110 120 130 140 150 160 170

(replib) Cyclohexanol, 5-methyl-2-(1-methylethyl)-, (1α,2β,5α)-(±)-

OH

Name: Cyclohexanol, 5-methyl-2-(1-methylethyl)-, (1α,2β,5α)-(±)- Formula: C10H20O

MW: 156 Exact Mass: 156.151415 CAS#: 15356-70-4 NIST#: 290746 ID#: 8667 DB: replib

Other DBs: TSCA, RTECS, HODOC, EINECS

Contributor: NIST Mass Spectrometry Data Center, 1998. 10 largest peaks:

71 999 | 81 916 | 95 796 | 55 544 | 41 525 | 82 409 | 123 380 | 67 368 | 43 362 | 57 361 |

Synonyms:

1.Menthol, (±)-

2.(±)-Menthol 3.DL-Menthol

4.Menthol, cis-1,3,trans-1,4-(±)-

5.Cyclohexanol, 5-methyl-2-(1-methylethyl)-, (1α,2β,5α)-(.+-.)- 6.Cyclohexanol, 5-methyl-2-(1-methylethyl)-,

7.4-Isopropyl-1-methylcyclohexan-3-ol 8.3-p-Menthol

9.dl-3-p-Menthanol 10.Menthol racemic 11.NCI-C50000

12.Menthol racemique 13.Menthol, (.+-.)-

14.(.+-.)-Menthol

15.Menthol, cis-1,3,trans-1,4-(.+-.)- 16.2-Isopropyl-5-methylcyclohexanol #
