# Supplementary material for: Effect of Mentha piperita Essential Oil and Its Nanoemulsion on Microbial Growth, Physicochemical, and Organoleptic Properties of Mango Yogurt During Refrigerated Storage
Source: Food Sci Nutr. 2026 May 1;14(5):e71845. doi: 10.1002/fsn3.71845 (PMC13135118; doi:10.1002/fsn3.71845)
Supplement: Supplementary file 2 — File S1: Supporting Information. [file FSN3-14-e71845-s002.zip › supplementary file 1/11.917.docx]

Hit 1 : Levomenthol

C10H20O; MF: 840; RMF: 891; Prob 18.9%; CAS: 2216-51-5; Lib: replib; ID: 10785.

81

71

41

95

HO

55

43

67

29

39

57

69

123

53

138

109

15

31

65

100

50

0

10 20 30 40 50 60 70 80 90 100 110 120 130 140 150 160 170

(replib) Levomenthol

HO

Name: Levomenthol Formula: C10H20O

MW: 156 Exact Mass: 156.151415 CAS#: 2216-51-5 NIST#: 114560 ID#: 10785 DB: replib

Other DBs: TSCA, RTECS, HODOC, NIH, EINECS, IRDB

Contributor: NIST Mass Spectrometry Data Center, 1990. Related CAS#: 98167-53-4

10 largest peaks:

81 999 | 71 936 | 95 849 | 41 811 | 55 619 | 67 546 | 43 509 | 82 386 | 69 369 | 57 354 |

Synonyms:

1.Cyclohexanol, 5-methyl-2-(1-methylethyl)-, [1R-(1α,2β,5α)]- 2.L-(-)-Menthol

3.Menthol, (1R,3R,4S)-(-)-

4.(-)-Menthol 5.(R)-(-)-Menthol

6.U.S.P. Menthol 7.1R-Menthol

8.(1R,2S,5R)-(-)-Menthol

9.(-)-Menthyl alcohol

10.(1R-(1-α,2-β,5-α))-5-Methyl-2-(1-methylethyl)cyclohexanol 11.2-Isopropyl-5-methylcyclohexanol-, (1R,2S,5R)- #

12.L-Menthol 13.(1R)-(-)-Menthol

14.(1R,2S,5R)-2-Isopropyl-5-methylcyclohexanol 15.Cyclohexanol, 5-methyl-2-(1-methylethyl)-, (1R,2S,5R)-

16.NSC 62788
