# Supplementary material for: Effect of Mentha piperita Essential Oil and Its Nanoemulsion on Microbial Growth, Physicochemical, and Organoleptic Properties of Mango Yogurt During Refrigerated Storage
Source: Food Sci Nutr. 2026 May 1;14(5):e71845. doi: 10.1002/fsn3.71845 (PMC13135118; doi:10.1002/fsn3.71845)
Supplement: Supplementary file 2 — File S1: Supporting Information. [file FSN3-14-e71845-s002.zip › supplementary file 1/13.201.docx]

Hit 1 : Cyclohexanone, 5-methyl-2-(1-methylethylidene)-

C10H16O; MF: 915; RMF: 919; Prob 41.9%; CAS: 15932-80-6; Lib: replib; ID: 10904.

81

67

O

152

109

41

39

137

29

43

33

53

51

69

95

65

79

91

124

100

50

0

20 30 40 50 60 70 80 90 100 110 120 130 140 150 160

(replib) Cyclohexanone, 5-methyl-2-(1-methylethylidene)-

O

Name: Cyclohexanone, 5-methyl-2-(1-methylethylidene)-Formula: C10H16O

MW: 152 Exact Mass: 152.120115 CAS#: 15932-80-6 NIST#: 190984 ID#: 10904 DB: replib

Other DBs: TSCA, RTECS, HODOC, EINECS, IRDB

Contributor: Chemical Concepts Related CAS#: 3285-04-9

10 largest peaks:

81 999 | 152 626 | 67 606 | 109 495 | 41 357 | 82 340 | 39 241 | 137 239 | 69 175 | 68 163 |

Synonyms:

1.p-Menth-4(8)-en-3-one 2.(±)-Pulegone

3.2-Isopropylidene-5-methylcyclohexanone 4.4(8)-p-Menthen-3-one

5.Pulegone

6.5-Methyl-2-(1-methylethylidene)cyclohexanone

Page 1 of 1
