# Supplementary material for: Effect of Mentha piperita Essential Oil and Its Nanoemulsion on Microbial Growth, Physicochemical, and Organoleptic Properties of Mango Yogurt During Refrigerated Storage
Source: Food Sci Nutr. 2026 May 1;14(5):e71845. doi: 10.1002/fsn3.71845 (PMC13135118; doi:10.1002/fsn3.71845)
Supplement: Supplementary file 2 — File S1: Supporting Information. [file FSN3-14-e71845-s002.zip › supplementary file 1/13.357.docx]

Hit 1 : cis-3-Hexenyl isovalerate

C11H20O2; MF: 747; RMF: 895; Prob 27.7%; CAS: 35154-45-1; Lib: replib; ID: 10974.

100 82

67

O

57

41 O

50 85

55

3943

73

0

92 103109115 124130 140 184

30 40 50 60 70 80 90 100 110 120 130 140 150 160 170 180 190

(replib) cis-3-Hexenyl isovalerate

O

O

Name: cis-3-Hexenyl isovalerate Formula: C11H20O2

MW: 184 Exact Mass: 184.14633 CAS#: 35154-45-1 NIST#: 122036 ID#: 10974 DB: replib

Other DBs: Fine, TSCA, RTECS, EINECS

Contributor: Dr. Jiri Zamecnik, DCIEM, North York, Canada, GP Library 10 largest peaks:

82 999 | 67 854 | 57 574 | 41 520 | 85 429 | 55 243 | 39 177 | 43 144 | 83 110 | 42 91 |

Synonyms:

1. Isovaleric acid cis-3-hexenyl ester
2. Butanoic acid, 3-methyl-, 3-hexenyl ester, (Z)- 3.Isovaleric acid, 3-hexenyl ester, (Z)-
3. AI3-35966
4. cis-3-Hexenyl 3-methylbutanoate 6.cis-3-Hexenyl iso-valerate

7.(3Z)-3-Hexenyl 3-methylbutanoate 8.(Z)-3-Hexenyl 3-methylbutanoate

9.(Z)-3-Hexenyl isopentanoate 10.(Z)-3-Hexenyl isovalerate
