# Supplementary material for: Effect of Mentha piperita Essential Oil and Its Nanoemulsion on Microbial Growth, Physicochemical, and Organoleptic Properties of Mango Yogurt During Refrigerated Storage
Source: Food Sci Nutr. 2026 May 1;14(5):e71845. doi: 10.1002/fsn3.71845 (PMC13135118; doi:10.1002/fsn3.71845)
Supplement: Supplementary file 2 — File S1: Supporting Information. [file FSN3-14-e71845-s002.zip › supplementary file 1/13.601.docx]

Hit 1 : 2-Cyclohexen-1-one, 3-methyl-6-(1-methylethyl)-

C10H16O; MF: 863; RMF: 907; Prob 67.3%; CAS: 89-81-6; Lib: replib; ID: 11043.

82

O

110

95

54

137

41

152

29

44

67

77

91

124

100

50

0

20 30 40 50 60 70 80 90 100 110 120 130 140 150 160

(replib) 2-Cyclohexen-1-one, 3-methyl-6-(1-methylethyl)-

O

Name: 2-Cyclohexen-1-one, 3-methyl-6-(1-methylethyl)- Formula: C10H16O

MW: 152 Exact Mass: 152.120115 CAS#: 89-81-6 NIST#: 77607 ID#: 11043 DB: replib

Other DBs: TSCA, RTECS, EPA, NIH, EINECS

Contributor: RESEARCH LABORATORY, STATE ALCOHOL MONOPOLY ALKO, HELSINKI, FINLAND; IRMA NYKANEN ET AL

10 largest peaks:

82 999 | 110 648 | 95 368 | 54 220 | 109 206 | 137 206 | 41 144 | 39 131 | 152 113 | 67 89 |

Synonyms:

1.p-Menth-1-en-3-one 2.Piperitone

3.3-Carvomenthenone

4.1-Methyl-4-isopropyl-1-cyclohexen-3-one 5.6-Isopropyl-3-methyl-2-cyclohexen-1-one #

Page 1 of 1
