# Supplementary material for: Effect of Mentha piperita Essential Oil and Its Nanoemulsion on Microbial Growth, Physicochemical, and Organoleptic Properties of Mango Yogurt During Refrigerated Storage
Source: Food Sci Nutr. 2026 May 1;14(5):e71845. doi: 10.1002/fsn3.71845 (PMC13135118; doi:10.1002/fsn3.71845)
Supplement: Supplementary file 2 — File S1: Supporting Information. [file FSN3-14-e71845-s002.zip › supplementary file 1/15.231.docx]

Hit 1 : Cyclohexanol, 5-methyl-2-(1-methylethyl)-, acetate

C12H22O2; MF: 957; RMF: 970; Prob 28.9%; CAS: 16409-45-3; Lib: replib; ID: 13457.

95

43

O

81

O

55

67

123

138

27

109

15

100

50

0

10 20 30 40 50 60 70 80 90 100 110 120 130 140 150 160 170 180 190 200 210

(replib) Cyclohexanol, 5-methyl-2-(1-methylethyl)-, acetate

O

O

Name: Cyclohexanol, 5-methyl-2-(1-methylethyl)-, acetate Formula: C12H22O2

MW: 198 Exact Mass: 198.16198 CAS#: 16409-45-3 NIST#: 114574 ID#: 13457 DB: replib

Other DBs: RTECS, EINECS, IRDB

Contributor: NIST Mass Spectrometry Data Center, 1990. 10 largest peaks:

95 999 | 43 768 | 81 676 | 138 344 | 41 326 | 123 302 | 55 296 | 67 280 | 82 272 | 96 217 |

Synonyms:

1. Menthol, acetate
2. Menthyl acetate
3. Acetic acid, p-menth-3-yl ester, dl- 4.dl-Menthyl acetate

5.Menthyl acetate racemic 6.l-Menthol acetate

7.2-Isopropyl-5-methylcyclohexyl acetate #

Page 1 of 1
