# Supplementary material for: Effect of Mentha piperita Essential Oil and Its Nanoemulsion on Microbial Growth, Physicochemical, and Organoleptic Properties of Mango Yogurt During Refrigerated Storage
Source: Food Sci Nutr. 2026 May 1;14(5):e71845. doi: 10.1002/fsn3.71845 (PMC13135118; doi:10.1002/fsn3.71845)
Supplement: Supplementary file 2 — File S1: Supporting Information. [file FSN3-14-e71845-s002.zip › supplementary file 1/18.455.docx]

Hit 1 : (-)-β-Bourbonene

C15H24; MF: 874; RMF: 888; Prob 78.3%; CAS: 5208-59-3; Lib: mainlib; ID: 45828.

81

123

161

41

27

55

91

105

33

67

133

147

189

204

100

50

0

20 30 40 50 60 70 80 90 100 110 120 130 140 150 160 170 180 190 200 210

(mainlib) (-)-β-Bourbonene

Name: (-)-β-Bourbonene Formula: C15H24

MW: 204 Exact Mass: 204.1878 CAS#: 5208-59-3 NIST#: 249537 ID#: 45828 DB: mainlib

Other DBs: NIH

Contributor: TNO Volatile Compounds in Food - Chemical Concepts 10 largest peaks:

81 999 | 80 737 | 123 574 | 161 249 | 79 209 | 41 175 | 105 105 | 91 95 | 77 84 | 55 78 |

Synonyms:

1. Cyclobuta[1,2:3,4]dicyclopentene, decahydro-3a-methyl-6-methylene-1-(1-methylethyl)-, [1S-(1α,3aα,3bβ,6aβ,6bα)

]-

1. β-Bourbonene
2. Cyclobuta[1,2:3,4]dicyclopentene, 1,2,3,3a,3bβ,4,5,6,6aβ,6bα-decahydro-1α-isopropyl-3aα-methyl-6-methylene- 4.Cyclobuta[1,2:3,4]dicyclopentene, decahydro-3a-methyl-6-methylene-1-(1-methylethyl)-, (1α,3aα,3bβ,6aβ,6bα)- 5.[1S-[1α,3aα,3bβ,6aβ,6bα]]-Cyclobuta[1,2:3,4]dicyclopentene-decahydro-3a-methyl-6-methylene-1-[1-methylethyl]

Page 1 of 1
