# Supplementary material for: Effect of Mentha piperita Essential Oil and Its Nanoemulsion on Microbial Growth, Physicochemical, and Organoleptic Properties of Mango Yogurt During Refrigerated Storage
Source: Food Sci Nutr. 2026 May 1;14(5):e71845. doi: 10.1002/fsn3.71845 (PMC13135118; doi:10.1002/fsn3.71845)
Supplement: Supplementary file 2 — File S1: Supporting Information. [file FSN3-14-e71845-s002.zip › supplementary file 1/19.50.docx]

Hit 1 : Caryophyllene

C15H24; MF: 941; RMF: 943; Prob 29.6%; CAS: 87-44-5; Lib: mainlib; ID: 60555.

100

93

133

41 69 79

105

50 120

55

161

147

27

0 15

175

189

204

10 20 30 40 50 60 70 80 90 100 110 120 130 140 150 160 170 180 190 200 210

(mainlib) Caryophyllene

Name: Caryophyllene Formula: C15H24

MW: 204 Exact Mass: 204.1878 CAS#: 87-44-5 NIST#: 291486 ID#: 60555 DB: mainlib

Other DBs: Fine, TSCA, RTECS, HODOC, NIH, EINECS

Contributor: NIST Mass Spectrometry Data Center, 1998. Related CAS#: 8007-38-3; 1407-53-0

10 largest peaks:

93 999 | 133 921 | 91 858 | 41 769 | 79 763 | 69 754 | 105 623 | 107 483 | 120 447 | 77 439 |

Synonyms:

1.Bicyclo[7.2.0]undec-4-ene, 4,11,11-trimethyl-8-methylene-, [1R-(1R*,4E,9S*)]- 2.Bicyclo[7.2.0]undec-4-ene, 4,11,11-trimethyl-8-methylene-, (E)-(1R,9S)-(-)- 3.β-Caryophyllen

4.β-Caryophyllene 5.trans-Caryophyllene 6.L-Caryophyllene

7.Bicyclo(7.2.0)undec-4-ene, 8-methylene-4,11,11-trimethyl-, (E)-(1R,9S)-(-)-

8.8-Methylene-4,11,11-(trimethyl)bicyclo(7.2.0)undec-4-ene, (1R,4E,9S)- 9.beta-Caryophyllene

10.β-(E)-Caryophyllene 11.β-trans-Caryophyllene 12.Caryophyllene, (E) 13.E-β-Caryophyllene 14.(E)-Caryophyllene 15.trans-β-Caryophyllene 16.(-)-(E)-Caryophyllene 17.Caryophyllene B

18.NSC 11906
