# Supplementary material for: Effect of Mentha piperita Essential Oil and Its Nanoemulsion on Microbial Growth, Physicochemical, and Organoleptic Properties of Mango Yogurt During Refrigerated Storage
Source: Food Sci Nutr. 2026 May 1;14(5):e71845. doi: 10.1002/fsn3.71845 (PMC13135118; doi:10.1002/fsn3.71845)
Supplement: Supplementary file 2 — File S1: Supporting Information. [file FSN3-14-e71845-s002.zip › supplementary file 1/2.710.docx]

Hit 1 : Tetrachloroethylene

C2Cl4; MF: 927; RMF: 963; Prob 96.8%; CAS: 127-18-4; Lib: replib; ID: 23696.

166

Cl

Cl

131

Cl

Cl

94

47

96

35

24

49

37 43

59

61

82

84

98

100

50

0

20 30 40 50 60 70 80 90 100 110 120 130 140 150 160 170 180

(replib) Tetrachloroethylene

Cl

Cl

Cl

Cl

Name: Tetrachloroethylene Formula: C2Cl4

MW: 164 Exact Mass: 163.875411 CAS#: 127-18-4 NIST#: 107150 ID#: 23696 DB: replib

Other DBs: Fine, TSCA, RTECS, EPA, USP, HODOC, NIH, EINECS, IRDB

Contributor: N.W. Davies, Centr. Sci. Lab., Univ. Tasmania, Hobart, Australia 10 largest peaks:

166 999 | 164 818 | 131 714 | 129 713 | 168 447 | 94 375 | 47 308 | 96 237 | 133 196 | 59 174 |

Synonyms:

1.Ethene, tetrachloro-2.Ethylene, tetrachloro-3.Ankilostin

4.Antisal 1 5.Didakene

6.Ethylene tetrachloride 7.Fedal-Un

8.Nema

9.Perchlorethylene 10.Perchloroethylene 11.Perclene 12.PerSec

13.Tetlen 14.Tetracap 15.Tetrachlorethylene 16.Tetrachloroethene 17.Tetraguer 18.Tetraleno 19.Tetropil

20.1,1,2,2-Tetrachloroethylene 21.C2Cl4

22.Carbon bichloride

23.Carbon dichloride 24.Czterochloroetylen

25.ENT 1,860

26.Nema, veterinary 27.NCI-C04580

28.Perawin 29.Perchloorethyleen, per 30.Perchloraethylen, per 31.Perchlorethylene, per 32.Percloroetilene 33.PERC

34.Tetrachlooretheen 35.Tetrachloraethen 36.Tetracloroetene 37.Tetralex

38.Antisol 1 39.Dow-per 40.Perchlor 41.Perclene D 42.Percosolve 43.PERK

44.Perklone

45.RCRA Waste Number U210 46.Tetravec

1. Tetroguer
2. UN 1897
3. Dilatin PT

50.1,1,2,2-Tetrachloroethene

51.Freon 1110 52.Perclene TG 53.Perchloroethene

54.F 1110
