# Supplementary material for: Effect of Mentha piperita Essential Oil and Its Nanoemulsion on Microbial Growth, Physicochemical, and Organoleptic Properties of Mango Yogurt During Refrigerated Storage
Source: Food Sci Nutr. 2026 May 1;14(5):e71845. doi: 10.1002/fsn3.71845 (PMC13135118; doi:10.1002/fsn3.71845)
Supplement: Supplementary file 2 — File S1: Supporting Information. [file FSN3-14-e71845-s002.zip › supplementary file 1/20.539.docx]

Hit 1 : 1,6,10-Dodecatriene, 7,11-dimethyl-3-methylene-

C15H24; MF: 817; RMF: 872; Prob 16.4%; CAS: 77129-48-7; Lib: mainlib; ID: 3250.

41

69

93

27

55

79

120

107

133

161

147

189

204

100

50

0

20 30 40 50 60 70 80 90 100 110 120 130 140 150 160 170 180 190 200 210

(mainlib) 1,6,10-Dodecatriene, 7,11-dimethyl-3-methylene-

Name: 1,6,10-Dodecatriene, 7,11-dimethyl-3-methylene-Formula: C15H24

MW: 204 Exact Mass: 204.1878 CAS#: 77129-48-7 NIST#: 151448 ID#: 3250 DB: mainlib

Other DBs: EINECS Contributor: Chemical Concepts 10 largest peaks:

41 999 | 69 898 | 93 379 | 39 296 | 55 233 | 67 222 | 79 217 | 53 210 | 27 208 | 81 153 |

Synonyms:

1.7,11-Dimethyl-3-methylene-1,6,10-dodecatriene
