# Supplementary material for: Effect of Mentha piperita Essential Oil and Its Nanoemulsion on Microbial Growth, Physicochemical, and Organoleptic Properties of Mango Yogurt During Refrigerated Storage
Source: Food Sci Nutr. 2026 May 1;14(5):e71845. doi: 10.1002/fsn3.71845 (PMC13135118; doi:10.1002/fsn3.71845)
Supplement: Supplementary file 2 — File S1: Supporting Information. [file FSN3-14-e71845-s002.zip › supplementary file 1/21.284.docx]

Hit 1 : 1,6-Cyclodecadiene, 1-methyl-5-methylene-8-(1-methylethyl)-, [S-(E,E)]- C15H24; MF: 918; RMF: 952; Prob 48.1%; CAS: 23986-74-5; Lib: mainlib; ID: 133132.

161

105

91

41

119

79

27

55

67

133

204

15

147

100

50

0

10 20 30 40 50 60 70 80 90 100 110 120 130 140 150 160 170 180 190 200 210

(mainlib) 1,6-Cyclodecadiene, 1-methyl-5-methylene-8-(1-methylethyl)-, [S-(E,E)]-

Name: 1,6-Cyclodecadiene, 1-methyl-5-methylene-8-(1-methylethyl)-, [S-(E,E)]- Formula: C15H24

MW: 204 Exact Mass: 204.1878 CAS#: 23986-74-5 NIST#: 221563 ID#: 133132 DB: mainlib

Other DBs: None

Contributor: P.A. Leclercq, Lab. Instrum. Anal., Tech. Univ. Eindhoven, Netherlands 10 largest peaks:

161 999 | 105 775 | 91 685 | 41 619 | 119 604 | 79 415 | 81 414 | 93 359 | 77 346 | 27 315 |

Synonyms:

1.Germacrene D

2.8-Isopropyl-1-methyl-5-methylene-1,6-cyclodecadiene, (1E,6E,8S)- 3.D-Germacrene

4.1(10),4(14),5-Germacratriene

Page 1 of 1
