# Supplementary material for: Effect of Mentha piperita Essential Oil and Its Nanoemulsion on Microbial Growth, Physicochemical, and Organoleptic Properties of Mango Yogurt During Refrigerated Storage
Source: Food Sci Nutr. 2026 May 1;14(5):e71845. doi: 10.1002/fsn3.71845 (PMC13135118; doi:10.1002/fsn3.71845)
Supplement: Supplementary file 2 — File S1: Supporting Information. [file FSN3-14-e71845-s002.zip › supplementary file 1/24.025.docx]

Hit 1 : Caryophyllene oxide

C15H24O; MF: 771; RMF: 884; Prob 29.2%; CAS: 1139-30-6; Lib: mainlib; ID: 5928.

43

79

93

O

55

69

109

27

121

135

149

161 177

187

205

220

100

50

0

20 30 40 50 60 70 80 90 100 110 120 130 140 150 160 170 180 190 200 210 220 230

(mainlib) Caryophyllene oxide

O

Name: Caryophyllene oxide Formula: C15H24O

MW: 220 Exact Mass: 220.182715 CAS#: 1139-30-6 NIST#: 156329 ID#: 5928 DB: mainlib

Other DBs: Fine, TSCA, RTECS, EINECS

Contributor: Chemical Concepts

Related CAS#: 105120-46-5; 11023-55-5; 32095-03-7; 52209-95-7

10 largest peaks:

43 999 | 41 927 | 79 885 | 93 661 | 91 573 | 95 420 | 69 407 | 55 393 | 67 377 | 81 373 |

Synonyms:

1.5-Oxatricyclo[8.2.0.0(4,6)-]dodecane, 4,12,12-trimethyl-9-methylene-, [1R-(1R*,4R*,6R*,10S*)]-2.5-Oxatricyclo(8.2.0.0(4,6))dodecane, 4,12,12-trimethyl-9-methylene-, (1R,4R,6R,10S)-3.Caryophylene oxide

4.Caryophyllene epoxide

5.(-)-β-Caryophyllene epoxide 6.β-Caryophyllene oxide 7.Epoxycaryophyllene

8.(-)-Epoxydihydrocaryophyllene

9.4,11,11-Trimethyl-8-methylene-5-oxatricyclo(8.2.0.0(4,6))dodecane, (1R,4R,6R,10S)-

10.(-)-5-Oxatricyclo[8.2.0.0(4,6)]dodecane,4,12,12-trimethyl-9-methylene-, [1R-(1R*,4R*,6R*,10S*)]-11.β-Caryophyllene epoxide

12.(-)-β-Caryophyllene oxide
