# Supplementary material for: Effect of Mentha piperita Essential Oil and Its Nanoemulsion on Microbial Growth, Physicochemical, and Organoleptic Properties of Mango Yogurt During Refrigerated Storage
Source: Food Sci Nutr. 2026 May 1;14(5):e71845. doi: 10.1002/fsn3.71845 (PMC13135118; doi:10.1002/fsn3.71845)
Supplement: Supplementary file 2 — File S1: Supporting Information. [file FSN3-14-e71845-s002.zip › supplementary file 1/24.385.docx]

Hit 1 : Ledol

C15H26O; MF: 861; RMF: 885; Prob 25.5%; CAS: 577-27-5; Lib: replib; ID: 1803.

43

OH

69

109

55

81

161

93

122

27

189

204

59

135 147

175

222

100

50

0

20 30 40 50 60 70 80 90 100 110 120 130 140 150 160 170 180 190 200 210 220 230

(replib) Ledol

OH

Name: Ledol Formula: C15H26O

MW: 222 Exact Mass: 222.198365 CAS#: 577-27-5 NIST#: 249593 ID#: 1803 DB: replib

Other DBs: HODOC

Contributor: TNO Volatile Compounds in Food - Chemical Concepts 10 largest peaks:

43 999 | 41 607 | 69 534 | 109 496 | 81 337 | 55 336 | 67 306 | 161 306 | 107 258 | 93 256 |

Synonyms:

1.1H-Cycloprop[e]azulen-4-ol, decahydro-1,1,4,7-tetramethyl-, [1ar-(1aα,4α,4aβ,7α,7aβ,7bα)]- 2.1H-Cycloprop[e]azulen-4-ol, decahydro-1,1,4,7-tetramethyl-, (1ar,4R,4as,7R,7as,7bs)-

3.(+)-Ledol 4.d-Ledol

5.1H-Cycloprop[e]azulen-4-ol, decahydro-1,1,4,7-tetramethyl-, [1ar-(1aα,4α,4aβ,7β,7aβ,7bα)]- 6.1,1,4,7-Tetramethyldecahydro-1H-cyclopropa[e]azulen-4-ol #

Page 1 of 1
