# Supplementary material for: Effect of Mentha piperita Essential Oil and Its Nanoemulsion on Microbial Growth, Physicochemical, and Organoleptic Properties of Mango Yogurt During Refrigerated Storage
Source: Food Sci Nutr. 2026 May 1;14(5):e71845. doi: 10.1002/fsn3.71845 (PMC13135118; doi:10.1002/fsn3.71845)
Supplement: Supplementary file 2 — File S1: Supporting Information. [file FSN3-14-e71845-s002.zip › supplementary file 1/4.894.docx]

Hit 1 : (1R)-2,6,6-Trimethylbicyclo[3.1.1]hept-2-ene

C10H16; MF: 927; RMF: 933; Prob 16.8%; CAS: 7785-70-8; Lib: replib; ID: 13150.

93

91

41

77

79

43

53

67

105

121

136

63

75

89

100

50

0

40 50 60 70 80 90 100 110 120 130 140 150

(replib) (1R)-2,6,6-Trimethylbicyclo[3.1.1]hept-2-ene

Name: (1R)-2,6,6-Trimethylbicyclo[3.1.1]hept-2-ene Formula: C10H16

MW: 136 Exact Mass: 136.1252 CAS#: 7785-70-8 NIST#: 140985 ID#: 13150 DB: replib

Other DBs: TSCA, EINECS

Contributor: Mark Whitten, Florida Museum of Natural History, U. of Florida 10 largest peaks:

93 999 | 91 454 | 92 440 | 77 358 | 41 325 | 79 270 | 53 135 | 105 118 | 121 115 | 80 111 |

Synonyms:

1.1R-α-Pinene

2.Bicyclo[3.1.1]hept-2-ene, 2,6,6-trimethyl-, (1R)-

3.2,6,6-Trimethylbicyclo[3.1.1]hept-2-ene-, (1R,5R)-4.d-α-Pinene

5.1R-(+)-α-Pinene

6.(R)-α-Pinene

7.α-Pinene, (D)-

Page 1 of 1
