# Supplementary material for: Effect of Mentha piperita Essential Oil and Its Nanoemulsion on Microbial Growth, Physicochemical, and Organoleptic Properties of Mango Yogurt During Refrigerated Storage
Source: Food Sci Nutr. 2026 May 1;14(5):e71845. doi: 10.1002/fsn3.71845 (PMC13135118; doi:10.1002/fsn3.71845)
Supplement: Supplementary file 2 — File S1: Supporting Information. [file FSN3-14-e71845-s002.zip › supplementary file 1/5.720.docx]

Hit 1 : β-Phellandrene

C10H16; MF: 912; RMF: 923; Prob 29.5%; CAS: 555-10-2; Lib: replib; ID: 13107.

93

77

91

41

79

27

29

43

53

57

65

136

69

105

115 121

100

50

0

20 30 40 50 60 70 80 90 100 110 120 130 140 150

(replib) β-Phellandrene

Name: β-Phellandrene Formula: C10H16

MW: 136 Exact Mass: 136.1252 CAS#: 555-10-2 NIST#: 151434 ID#: 13107 DB: replib

Other DBs: TSCA, HODOC, NIH, EINECS

Contributor: Chemical Concepts 10 largest peaks:

93 999 | 77 298 | 91 274 | 79 183 | 41 162 | 39 152 | 94 119 | 136 108 | 27 93 | 80 75 |

Synonyms:

1.Cyclohexene, 3-methylene-6-(1-methylethyl)-2.p-Mentha-1(7),2-diene

3.Phellandrene, β

4.3-Isopropyl-6-methylene-1-cyclohexene # 5.beta-Phellandrene

6.3-methylene-6-(1-methylethenyl)-cyclohexane

Hit 2 : Bicyclo[3.1.0]hexane, 4-methylene-1-(1-methylethyl)-

C10H16; MF: 909; RMF: 939; Prob 26.1%; CAS: 3387-41-5; Lib: replib; ID: 13040.

93

27

29

41

39

43

69

77

79

91

53

65

136

105

121

100

50

0

20 30 40 50 60 70 80 90 100 110 120 130 140 150

(replib) Bicyclo[3.1.0]hexane, 4-methylene-1-(1-methylethyl)-

Name: Bicyclo[3.1.0]hexane, 4-methylene-1-(1-methylethyl)-Formula: C10H16

MW: 136 Exact Mass: 136.1252 CAS#: 3387-41-5 NIST#: 34194 ID#: 13040 DB: replib

Other DBs: Fine, NIH, EINECS

Contributor: W. UTILIZATION R & D DIV., U.S. DEPT. OF AGRIC., ALBANY, CAL.

10 largest peaks:

93 999 | 41 278 | 77 242 | 91 198 | 79 179 | 27 174 | 39 174 | 69 132 | 94 103 | 136 81 |

Synonyms:

1.4(10)-Thujene 2.Sabinen 3.Sabinene 4.(+)-Sabinene

5.THUJENE, 4(10)-

6.1-Isopropyl-4-methylenebicyclo[3.1.0]hexane 7.Sabenene

8.NSC 407278
