# Supplementary material for: Effect of Mentha piperita Essential Oil and Its Nanoemulsion on Microbial Growth, Physicochemical, and Organoleptic Properties of Mango Yogurt During Refrigerated Storage
Source: Food Sci Nutr. 2026 May 1;14(5):e71845. doi: 10.1002/fsn3.71845 (PMC13135118; doi:10.1002/fsn3.71845)
Supplement: Supplementary file 2 — File S1: Supporting Information. [file FSN3-14-e71845-s002.zip › supplementary file 1/5.837.docx]

Hit 1 : β-Pinene

C10H16; MF: 935; RMF: 936; Prob 28.8%; CAS: 127-91-3; Lib: mainlib; ID: 59836.

100 93

41

50

39

27

29 43

15

0

69

77 91

53 67

55

58 74 89

107

121

136

10 20 30 40 50 60 70 80 90 100 110 120 130 140 150

(mainlib) β-Pinene

Name: β-Pinene Formula: C10H16

MW: 136 Exact Mass: 136.1252 CAS#: 127-91-3 NIST#: 118895 ID#: 59836 DB: mainlib

Other DBs: TSCA, RTECS, NIH, EINECS, IRDB

Contributor: NIST Mass Spectrometry Data Center, 1990. Related CAS#: 23089-32-9

10 largest peaks:

93 999 | 41 609 | 69 350 | 39 318 | 91 308 | 77 275 | 79 274 | 27 214 | 92 152 | 53 134 |

Synonyms:

1.Bicyclo[3.1.1]heptane, 6,6-dimethyl-2-methylene- 2.2(10)-Pinene

3.Nopinen 4.Nopinene 5.Pseudopinen 6.Pseudopinene 7.Terebenthene

8.6,6-Dimethyl-2-methylene-bicyclo[3.1.1]heptane 9.Pinene, β

10.2,2,6-Trimethylbicyclo(3.1.1)hept-2-ene 11.Bicyclo[3.1.1]heptane-6,6-trimethyl, 2-methylene 12.(±)-β-Pinene

1. NSC 21447
2. beta-Pinene
