# Supplementary material for: Effect of Mentha piperita Essential Oil and Its Nanoemulsion on Microbial Growth, Physicochemical, and Organoleptic Properties of Mango Yogurt During Refrigerated Storage
Source: Food Sci Nutr. 2026 May 1;14(5):e71845. doi: 10.1002/fsn3.71845 (PMC13135118; doi:10.1002/fsn3.71845)
Supplement: Supplementary file 2 — File S1: Supporting Information. [file FSN3-14-e71845-s002.zip › supplementary file 1/6.138.docx]

Hit 1 : β-Myrcene

C10H16; MF: 783; RMF: 808; Prob 32.9%; CAS: 123-35-3; Lib: mainlib; ID: 3577.

100 41

93

69

50

27

29

15 32

0

39

53

43 51 55

67 79 91

74 89

107 121 136

10 20 30 40 50 60 70 80 90 100 110 120 130 140 150

(mainlib) β-Myrcene

Name: β-Myrcene Formula: C10H16

MW: 136 Exact Mass: 136.1252 CAS#: 123-35-3 NIST#: 3301 ID#: 3577 DB: mainlib

Other DBs: Fine, TSCA, RTECS, HODOC, NIH, EINECS, IRDB Related CAS#: 2153-31-3

10 largest peaks:

41 999 | 93 855 | 69 796 | 39 299 | 27 280 | 53 141 | 79 138 | 67 110 | 77 110 | 91 95 |

Synonyms:

1.1,6-Octadiene, 7-methyl-3-methylene-2.Myrcene

3.7-Methyl-3-methylene-1,6-octadiene 4.7-Methyl-3-methyleneoctadiene-(1,6) 5.2-Methyl-6-methylene-2,7-octadiene 6.3-Methylene-7-methyl-1,6-octadiene 7.β-Geraniolene

8.7-Methyl-3-methylene-octa-1,6-diene

9.beta-Myrcene 10.NSC 406264
