# Supplementary material for: Effect of Mentha piperita Essential Oil and Its Nanoemulsion on Microbial Growth, Physicochemical, and Organoleptic Properties of Mango Yogurt During Refrigerated Storage
Source: Food Sci Nutr. 2026 May 1;14(5):e71845. doi: 10.1002/fsn3.71845 (PMC13135118; doi:10.1002/fsn3.71845)
Supplement: Supplementary file 2 — File S1: Supporting Information. [file FSN3-14-e71845-s002.zip › supplementary file 1/6.931.docx]

Hit 1 : o-Cymene

C10H14; MF: 903; RMF: 930; Prob 24.1%; CAS: 527-84-4; Lib: mainlib; ID: 88975.

119

91

134

39

65

77

51

15

27

58

74

89

103

100

50

0

10 20 30 40 50 60 70 80 90 100 110 120 130 140

(mainlib) o-Cymene

Name: o-Cymene Formula: C10H14

MW: 134 Exact Mass: 134.10955 CAS#: 527-84-4 NIST#: 114006 ID#: 88975 DB: mainlib

Other DBs: Fine, TSCA, RTECS, EPA, HODOC, NIH, EINECS, IRDB

Contributor: NIST Mass Spectrometry Data Center, 1990. 10 largest peaks:

119 999 | 91 393 | 134 256 | 117 160 | 77 127 | 65 125 | 115 110 | 39 101 | 120 98 | 51 78 |

Synonyms:

1.Benzene, 1-methyl-2-(1-methylethyl)- 2.o-Cymol

3.o-Isopropyltoluene

4.1-Isopropyl-2-methylbenzene 5.1-Methyl-2-isopropylbenzene 6.2-Isopropyltoluene

7.1-Methyl-2-(1-methylethyl)-benzene 8.Cymene, ortho

9.UN 2046

10.1-(1-methylethyl)-2-methylbenzene 11.1-methyl,2-n-isopropylbenzene 12.ortho-Cymene

Hit 2 : Benzene, 1-methyl-3-(1-methylethyl)-

C10H14; MF: 897; RMF: 924; Prob 18.9%; CAS: 535-77-3; Lib: replib; ID: 17252.

119

91

134

39

51

65

77

15

27 32

58

103

100

50

0

10 20 30 40 50 60 70 80 90 100 110 120 130 140

(replib) Benzene, 1-methyl-3-(1-methylethyl)-

Name: Benzene, 1-methyl-3-(1-methylethyl)- Formula: C10H14

MW: 134 Exact Mass: 134.10955 CAS#: 535-77-3 NIST#: 113968 ID#: 17252 DB: replib

Other DBs: Fine, TSCA, RTECS, EPA, HODOC, NIH, EINECS, IRDB

Contributor: NIST Mass Spectrometry Data Center, 1990. 10 largest peaks:

119 999 | 91 357 | 134 237 | 117 147 | 65 113 | 77 110 | 120 101 | 39 93 | 115 89 | 51 73 |

Synonyms:

1.m-Cymene 2.β-Cymene 3.m-Cymol

4.m-Isopropyltoluene

5.m-Methylisopropylbenzene 6.1-Isopropyl-3-methylbenzene 7.1-Methyl-3-isopropylbenzene

8.1-Methyl-3-(1-methylethyl)-benzene

9.1-Methyl-3-isopropyl benzene 10.3-Methyl-1-isopropylbenzene 11.meta-Cymene

12.3-Isopropyltoluene

13.NSC 73975

14.UN 2046 (Related)
