# Supplementary material for: Effect of Mentha piperita Essential Oil and Its Nanoemulsion on Microbial Growth, Physicochemical, and Organoleptic Properties of Mango Yogurt During Refrigerated Storage
Source: Food Sci Nutr. 2026 May 1;14(5):e71845. doi: 10.1002/fsn3.71845 (PMC13135118; doi:10.1002/fsn3.71845)
Supplement: Supplementary file 2 — File S1: Supporting Information. [file FSN3-14-e71845-s002.zip › supplementary file 1/7.181.docx]

Hit 1 : Limonene

C10H16; MF: 933; RMF: 937; Prob 23.4%; CAS: 138-86-3; Lib: replib; ID: 7873.

100 68

39

50 41

27

29

0 32

53

51 55 65

58

93

79

77

75 89

107

121 136

20 30 40 50 60 70 80 90 100 110 120 130 140 150

(replib) Limonene

Name: Limonene Formula: C10H16

MW: 136 Exact Mass: 136.1252 CAS#: 138-86-3 NIST#: 64032 ID#: 7873 DB: replib

Other DBs: TSCA, RTECS, EPA, HODOC, NIH, EINECS, IRDB

Contributor: D.HENNEBERG, MAX-PLANCK INSTITUTE, MULHEIM, WEST GERMANY Related CAS#: 555-08-8; 7705-14-8; 8050-32-6

10 largest peaks:

68 999 | 67 637 | 93 603 | 39 578 | 41 463 | 53 414 | 79 407 | 27 397 | 94 278 | 77 261 |

Synonyms:

1.Cyclohexene, 1-methyl-4-(1-methylethenyl)-2.p-Mentha-1,8-diene

3.α-Limonene 4.Cajeputen 5.Cajeputene 6.Cinen 7.Cinene

8.Dipenten 9.Dipentene 10.Eulimen 11.Kautschin 12.Limonen 13.Nesol

14.p-Mentha-1,8(9)-diene 15.δ-1,8-Terpodiene

16.p-Mentha-1,8-diene, dl-17.Acintene DP dipentene 18.Di-p-mentha-1,8-diene 19.DL-Limonene 20.Inactive limonene

21.1-Methyl-4-isopropenyl-1-cyclohexene 22.1,8(9)-p-Menthadiene

23.4-Isopropenyl-1-methyl-1-cyclohexene 24.Acintene DP

1. Dipanol
2. UN 2052
3. Unitene

28.4-Isopropenyl-1-methylcyclohexene 29.1,8-p-Menthadiene

30.1-Methyl-4-(1-methylethenyl)cyclohexene 31.1-Methyl-4-isopropenylcyclohexene 32.(±)-Limonene

33.Achilles dipentene

34.1-methyl-4-isopropenylcyclohex-1-ene 35.1-methyl-4-(prop-1-en-2-yl)cyclohex-1-ene 36.Cyclohexene, 1-methyl-4-(1-methylethynyl)

37.Cyclohexene, 1-methyl-4-(1-methylethenyl)-, (±)-38.(±)-Dipentene

39.(±)-α-Limonene 40.Flavor orange 41.Goldflush II

1. NSC 21446
2. Orange flavor
3. PC 560
