# Supplementary material for: Effect of Mentha piperita Essential Oil and Its Nanoemulsion on Microbial Growth, Physicochemical, and Organoleptic Properties of Mango Yogurt During Refrigerated Storage
Source: Food Sci Nutr. 2026 May 1;14(5):e71845. doi: 10.1002/fsn3.71845 (PMC13135118; doi:10.1002/fsn3.71845)
Supplement: Supplementary file 2 — File S1: Supporting Information. [file FSN3-14-e71845-s002.zip › supplementary file 1/8.137.docx]

Hit 1 : Cyclohexanol, 1-methyl-4-(1-methylethenyl)-, cis-

C10H18O; MF: 898; RMF: 937; Prob 48.0%; CAS: 7299-41-4; Lib: mainlib; ID: 8311.

43

OH

41

71

55

93

45

53

51

69

67

81

111

58

77

83

121

86

97

139

107

125

154

100

50

0

40 50 60 70 80 90 100 110 120 130 140 150 160

(mainlib) Cyclohexanol, 1-methyl-4-(1-methylethenyl)-, cis-

OH

Name: Cyclohexanol, 1-methyl-4-(1-methylethenyl)-, cis-Formula: C10H18O

MW: 154 Exact Mass: 154.135765 CAS#: 7299-41-4 NIST#: 140973 ID#: 8311 DB: mainlib

Other DBs: None

Contributor: Mark Whitten, Florida Museum of Natural History, U. of Florida 10 largest peaks:

43 999 | 71 534 | 41 500 | 55 335 | 93 316 | 69 240 | 81 230 | 111 183 | 79 171 | 67 142 |

Synonyms:

1.Terpineol, cis-β-

2.p-Menth-8-en-1-ol, cis 3.cis-β-Terpineol

4.cis-p-Menth-8-en-1-ol 5.(Z)-β-Terpineol

6.4-Isopropenyl-1-methylcyclohexanol, cis

Page 1 of 1
