# Supplementary material for: Effect of Mentha piperita Essential Oil and Its Nanoemulsion on Microbial Growth, Physicochemical, and Organoleptic Properties of Mango Yogurt During Refrigerated Storage
Source: Food Sci Nutr. 2026 May 1;14(5):e71845. doi: 10.1002/fsn3.71845 (PMC13135118; doi:10.1002/fsn3.71845)
Supplement: Supplementary file 2 — File S1: Supporting Information. [file FSN3-14-e71845-s002.zip › supplementary file 1/Nana.docx]

Plot 1. 020129 nana-davati3.xms - 4/18/2023 1:37 PM

Lock Peak Width: No

Parameters: Local

Peak Width (sec): 4.0

Slope Sensitivity (SN): 10

Tangent %: 10

Peak Size Reject (counts): 18000000

Smoothing: Mean 3 Point Smooth

Spike Threshold Factor: 10

Noise: Peak to Peak

| Retention Time | Area | % of Total | Signal/Noise | Scan Description |
| --- | --- | --- | --- | --- |
| 1. 2.710 | 2.765e+7 | 0.135 | 62.2 | Merged |
| 2. 4.894 | 1.133e+8 | 0.555 | 382.2 | Merged |
| 3. 5.720 | 5.366e+7 | 0.263 | 154.9 | Merged |
| 4. 5.837 | 1.307e+8 | 0.640 | 372.6 | Merged |
| 5. 6.138 | 5.167e+7 | 0.253 | 129.2 | Merged |
| 6. 6.931 | 3.034e+7 | 0.149 | 38.86 | Merged |
| 7. 7.181 | 1.000e+9 | 4.898 | 1548 | Merged |
| 8. 8.137 | 2.137e+7 | 0.105 | 56.42 | Merged |
| 9. 10.577 | 3.471e+9 | 16.999 | 3126 | Merged |
| 10. 10.845 | 5.720e+8 | 2.802 | 83.99 | Merged |
| 11. 11.055 | 1.583e+9 | 7.755 | 80.06 | Merged |
| 12. 11.181 | 8.462e+8 | 4.144 | 25.29 | Merged |
| 13. 11.487 | 9.767e+9 | 47.837 | 572.9 | Merged |
| 14. 11.764 | 8.017e+7 | 0.393 | 14.58 | Merged |
| 15. 11.917 | 4.240e+7 | 0.208 | 16.97 | Merged |
| 16. 13.201 | 6.365e+8 | 3.117 | 477.6 | Merged |
| 17. 13.357 | 9.011e+7 | 0.441 | 79.02 | Merged |
| 18. 13.601 | 1.053e+8 | 0.516 | 106.1 | Merged |
| 19. 14.675 | 6.568e+7 | 0.322 | 94.09 | Merged |
| 20. 15.231 | 1.141e+9 | 5.588 | 1914 | Merged |
| 21. 15.697 | 3.700e+7 | 0.181 | 50.47 | Merged |
| 22. 18.455 | 3.565e+7 | 0.175 | 63.52 | Merged |
| 23. 19.500 | 3.025e+8 | 1.482 | 583.4 | Merged |
| 24. 20.539 | 3.202e+7 | 0.157 | 27.78 | Merged |
| 25. 21.284 | 1.232e+8 | 0.603 | 164 | Merged |
| 26. 24.025 | 3.222e+7 | 0.158 | 103.2 | Merged |
| 27. 24.385 | 2.563e+7 | 0.126 | 60.91 | Merged |
